# Mechanism of *Borrelia* immune evasion by FhbA-related proteins

**DOI:** 10.1371/journal.ppat.1010338

**Published:** 2022-03-18

**Authors:** Konstantin Kogan, Karita Haapasalo, Tommi Kotila, Robin Moore, Pekka Lappalainen, Adrian Goldman, Taru Meri

**Affiliations:** 1 HiLife Institute of Biotechnology, University of Helsinki, Helsinki, Finland; 2 Department of Bacteriology and Immunology, University of Helsinki, Helsinki, Finland; 3 Faculty of Biological and Environmental Sciences, University of Helsinki, Helsinki, Finland; 4 Astbury Center for Structural Molecular Biology, University of Leeds, Leeds, United Kingdom; Medical College of Wisconsin, UNITED STATES

## Abstract

Immune evasion facilitates survival of *Borrelia*, leading to infections like relapsing fever and Lyme disease. Important mechanism for complement evasion is acquisition of the main host complement inhibitor, factor H (FH). By determining the 2.2 Å crystal structure of Factor H binding protein A (FhbA) from *Borrelia hermsii* in complex with FH domains 19–20, combined with extensive mutagenesis, we identified the structural mechanism by which *B*. *hermsii* utilizes FhbA in immune evasion. Moreover, structure-guided sequence database analysis identified a new family of FhbA-related immune evasion molecules from Lyme disease and relapsing fever *Borrelia*. Conserved FH-binding mechanism within the FhbA-family was verified by analysis of a novel FH-binding protein from *B*. *duttonii*. By sequence analysis, we were able to group FH-binding proteins of *Borrelia* into four distinct phyletic types and identified novel putative FH-binding proteins. The conserved FH-binding mechanism of the FhbA-related proteins could aid in developing new approaches to inhibit virulence and complement resistance in *Borrelia*.

## Author summary

Relapsing fever and Lyme Disease are infectious diseases caused by borrelia bacteria. Relapsing fever occurs sporadically worldwide, whereas distribution of Lyme Disease is restricted to the Northern Hemisphere. Both infections are transmitted to humans by blood eating ticks or lice. These infections are often difficult to diagnose due to nonspecific symptoms. To be able to cause infection, borrelia must circumvent the human immune responses. Here we describe a mechanism, how borrelia bacteria protect themselves in the human host by utilizing host proteins. By using X-ray crystallography, we solved the structure of an outer membrane protein FhbA from a relapsing fever causing borreliae, *Borrelia hermsii*, in complex with human complement regulator factor H. FhbA has a unique alpha-helical fold that has not been reported earlier. The structure of the complex revealed how FhbA binds factor H in a very specific manner. Factor H bound to FhbA on the surface of borrelia protects bacteria from the complement system and lysis. Based on the structure, we performed structure-guided sequence database analysis, which suggests that similar proteins are present in all relapsing fever causing borrelia and possibly in some Lyme disease agents.

## Introduction

Lyme disease (LD) and relapsing fever (RF) are infections caused by borreliae spirochetes. Bacteria are transmitted to humans from vertebrate animals by blood feeding arthropods. RF and LD borreliae have different vectors. All LD borreliae are transmitted by *Ixodes* ticks and the majority of RF borreliae by soft bodied *Ornithodoros* ticks. Borreliae differ also in their outer membrane composition, genomic features, selection of vertebrate hosts, and immune evasion mechanisms [1]. Due to differences between the two groups, the division of genus *Borrelia* into two was recently suggested: RF-causing species would constitute the genus *Borrelia*, and LD-causing species the genus *Borreliella* [2]. In this publication, we chose to use the most recent nomenclature, even though the scientific community has not reached full consensus on the subject [3].

Lyme disease is mainly caused by three different genospecies of *Borreliella* (*Bo*. *burgdorferi*, *Bo*. *afzelii* and *Bo*. *garinii*). *Bo*. *burgdorferi* is found in North America, whereas all three exist in Europe [4]. In the US there are 42,000 (https://www.cdc.gov/lyme/) and in Europe 70,000 cases of LD reported each year [5]. In LD, bacteria initially affect skin around the tick bite causing the most common sign of the infection, a ring-like rash called erythema migrans. Systemic manifestations of disseminated infection include neuroborreliosis, carditis and arthritis [6].

Relapsing fever is caused by many species of borreliae that are present worldwide in warm and temperate regions [7]. Individual species have restricted geographical distributions. In the US, RF outbreaks are mainly caused by B. hermsii and B. turicatae [8]; whereas in Africa they are caused by B. duttonii and B. crocidurae [9]. Clinically, RF is characterized by recurring high fever episodes accompanied by nonspecific symptoms. The reason for recurrent fever episodes is antigenic variation: the ability of RF borrelia to change variable outer surface proteins to evade antibody recognition. Neurological manifestations, myocarditis and development of organ failure in kidneys, spleen and lungs in severe cases may follow [10,11]. Without adequate treatment, mortalities of up to 5% in epidemics of tick-borne RF have been described [12].

The alternative pathway (AP) of the complement system is the first innate immune defense mechanism targeting microbes. Activation begins by attachment of C3b proteins to the surface of a microbe. Surface-bound C3b-molecules form the basis for complement amplification leading to formation of the lytic membrane attack complexes. Complement activation needs to be tightly controlled to prevent harmful effects on the host. Many complement proteins are regulators of the system on surfaces and in the fluid phase (for a review, see [13]). The main AP regulator in serum is Factor H (FH), which consists of 20 globular short consensus repeat domains called SCR or FH-domains. FH regulates AP efficiently by three different mechanisms, all of which require that domains 1–4 of FH are bound to C3b [14–16].

In the complement system, there are also FH-related proteins. Factor H-like protein 1 (FHL-1) is an alternatively spliced transcript of the *cfh* gene. FHL-1 consists of FH domains 1–7 with a unique C-terminal tail of four amino acids, and it has similar regulatory functions as FH [14]. In addition, there are five Factor H-related proteins (FHR1-5) encoded by their own genes. They contain four to nine SCR-domains, some with high homology to FH-domains [17]. The exact functions of FHRs are presently not known, but they may regulate FH by preventing its binding to ligands [18].

For extracellular microbes, evasion of complement is a prerequisite for infectivity. Spirochetes as diderm bacteria are structurally vulnerable to complement mediated lysis [19], thus for them complement evasion is a fundamental requirement. During a blood meal in the vector’s midgut, and during the dissemination phase *via* skin and blood in humans, borreliae are unavoidably in direct contact with the complement system.

One of the best characterized complement evasion mechanisms, in spirochetes as in other microbes, is the utilization of FH. In LD-causing borreliae, several FH-binding proteins have been described (reviewed in [20]). Proteins can be grouped into three different classes by sequence similarity. First class is CspA-related proteins, where the canonical protein is CspA (or BBA68, Complement regulator-acquiring surface protein, BbCRASP-1) from *Bo*. *burgdorferi*. The second entity is the CspZ-related proteins, where the canonical protein is CspZ (BBH06/BbCRASP-2) from *Bo*. *burgdorferi*. Both groups of proteins bind FH and FHL-1. The third group is very large, as it contains several proteins from the OspE/OspF-related family. All proteins, which have been analysed in detail from the third group bind FH *via* the common microbial binding site on domain 20 of FH. Structures of CspA [21], CspZ [2], as well as OspE [22] and its homologues ErpC and ErpP [23] have been determined. The crystal structure of the OspE:FH19-20 complex showed in detail how OspeE bound FH [22].

In addition to binding FH, several other complement avoidance mechanisms have been described in borreliae (reviewed *e*.*g*., in [1]). For example, *Bo*. *burgdorferi* proteins BBK32 [24,25] and OspC [26] prevent classical pathway activation, and BGA66 and BGA71 of *Bo*. *bavarensis* inhibit the terminal pathway [27].

Binding of FH has also been described for RF agents [28], and a ligand for FH in *B*. *hermsii* was shown by Hovis *et al*. to be a plasmid-encoded, surface-exposed Factor H binding protein A (FhbA)[29]. FhbA from *B*. *hermsii* (BhFhbA) was previously shown to interact with FH *via* domain 20 [30] and it was also reported to bind FHL-1 [31]. Other FH-binding proteins described from RF spirochetes include BpcA from *B*. *parkeri* [32], HcpA from *B*. *recurrentis* [33] and CbiA from *B*. *miyamotoi* [34]. However, the mechanisms by which these proteins bind FH have remained elusive.

By applying X-ray crystallography, we determined the structure of FhbA from *B*. *hermsii* in complex with its main ligand, FH domains 19–20, revealing a novel fold. The binding mechanism obtained from our co-crystal structure was confirmed by structure-guided mutagenesis and subsequent binding assays. We show that serum sensitive *E*. *coli* expressing functional wild type FhbA protein on the outer membrane surface were protected against complement, whereas bacteria expressing mutant FhbA protein unable to bind FH were as sensitive to complement as *E*. *coli*. We also demonstrated that the ability of FhbA to bind FH through this structural mechanism is important in complement evasion of live *B*. *hermsii* in a whole blood assay. Finally, by sequence comparisons and secondary structure predictions, we identified a putative new family of FH-binding proteins in both RF and LD borreliae. By analysing a previously uncharacterized member of this family from *B*. *duttonii* (BdFhbA), we verified the conservation of the unique FH-binding fold.

## Results

### 2.2 Å crystal structure of *Borrelia hermsii* FhbA in complex with factor H domains 19–20

To understand how FH binds to the microbial surface protein BhFhbA, we determined the crystal structure of BhFhbA in complex with the FH19-20 fragment at 2.2 Å resolution. The initial solution was obtained by molecular replacement with the published structure of FH19-20 (PDB 2G7I [35]) as a search model. We identified a single molecule of FH19-20 in the asymmetric unit with clear density for another polypeptide. Next, we assigned BhFhbA to the density by multiple rounds of manual building in Coot [36] and refinement with BUSTER [37]. The final solution showed a 1:1 complex of FH19-20 and BhFhbA in the asymmetric unit. The model was refined to a good overall geometry, and water molecules and ions were introduced to clear unassigned densities. The final model fits the observed diffraction data with the final R_work_/R_free_ values of 19.7%/23.7% (Table 1), and all residues are in the most favored (98.0%) or allowed (1.65%) regions of the Ramachandran plot.

**Table 1:** Crystallographic data and model quality.

	BhFhbA:FH19-20 complex
Wavelength	0.87290Å
Resolution range (highest resolution in parentheses)	37.83–2.2 (2.24–2.2)
Space group	P 2_1_2_1_2_1_
Unit cell (Å)	43.27 60.33 145.68
Total reflections	264765 (13683)
Unique reflections	20146 (993)
Multiplicity	13.1 (13.8)
Completeness (%)	100.00 (99.00)
Mean I/σ(I)	12.53 (1.04)
Wilson B-factor (Å^2^)	43.58
R-merge (%)	0.166 (2.44)
R-meas (%)	0.172 (2.53)
R-pim (%)	0.047 (0.673)
CC_1/2_	0.999 (0.513)
CC*	1 (0.831)
Reflections used in refinement	20103 (1981)
Reflections used for R-free	984 (96)
R-work	0.197 (0.3748)
R-free	0.237 (0.4500)
CC(work)	0.959 (0.773)
CC(free)	0.950 (0.593)
Number of non-hydrogen atoms	2543
macromolecules	2348
ligands	41
solvent	154
Protein residues	285
RMS (bonds) (Å)	0.013
RMS (angles) (°)	1.67
Ramachandran favored (%)	98.0
Ramachandran allowed (%)	1.65
Ramachandran outliers (%)	0.35
Rotamer outliers (%)	0.35
Clashscore	1.69
MolProbity score	0.95
Average B-factor (Å^2^)	59.90
macromolecules	59.10
ligands	100.7
solvent	61.50

**Fig 1:**
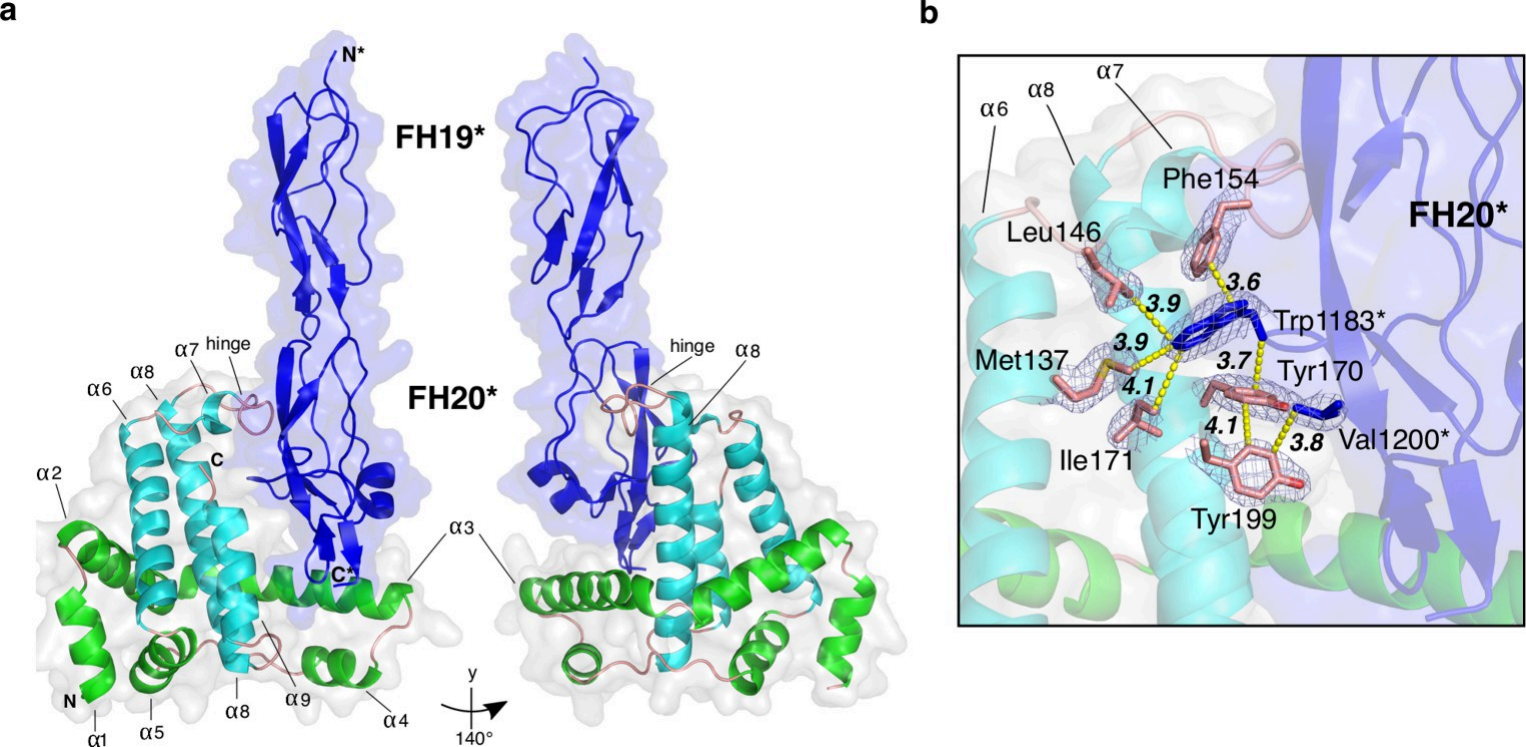
2.2 Å crystal structure of BhFhbA in complex with FH19-20. (a) A cartoon representation of the BhFhbA:FH19-20 complex. BhFhbA forms a bundle of nine α-helices resembling an arc-like or L-shaped structure. α-helices 1–5 (in green) wrap around the core formed by α-helices 6–9 (in teal). The connecting loop of α-helices 7 and 8 create a hinge-like structure close to C-terminus of BhFhbA. Together the L-shaped cavity (α-helices 3, 8 and 9) and the hinge-loop coordinate the binding of FH20 (in blue). (b) Close-up of the binding site with key sidechains represented as sticks. FH20 Trp1183 and Val1200 (in blue, indicated by *) coordinate binding by a group of hydrophobic residues in BhFhbA. Trp1183 of FH20 is inserted to the hydrophobic pocket formed by BhFhbA Phe154, Met137, I171 and Tyr170. Further, FH20 Val1200 is within van der Waals distance of BhFhbA Tyr170 and Tyr199. 2Fo-Fc density, contoured at 1 σ, is shown around the residues.

The structure of BhFhbA (residues 44–202) reveals a compact, single-domain fold, which is composed solely of a bundle of nine α-helices (Fig 1A). The first five α-helices (α1-α5) wrap around the core formed by α-helices 6–9 with the overall architecture of the fold resembling a curved arc or capital letter ‘L’. Helices 6–9 are essentially perpendicular to the rest of the helices. α3, α6, α8 and α9 compose the inside face of the L, creating a cavity with a hinge-like loop on top, formed by the connecting region between α-helices 6 and 7.

We performed a structure-guided search from the Protein Data Bank to analyze the conservation of the protein. Interestingly, we could not find any proteins with a similar fold either from PDBeFold or the Dali server [38]. The structure of BhFhbA is thus a previously uncharacterized fold and further represents a novel FH-binding scaffold.

There are two possible FH19-20-BhFhbA interfaces in the crystal structure (S1 Fig). The primary and biologically relevant interface in the FH20 domain has a buried surface area of 1066 Å^2^, as determined by the PISA server [39]. The other interface, located at the tip of domain 20 of FH, arises from crystal packing. Further, the primary, biologically relevant interface contains more specific contacts between FH and FhbA, as demonstrated by the increased number of hydrogen bonds when compared to the other interface (18 vs. 6, S1 Table).

A careful analysis of the primary interface reveals that α-helices 6–9 of BhFhbA form a hydrophobic cavity in which FH20 sits (Fig 1B). Within the cavity, Trp1183 of FH20 surrounded by a sandwich like stack of aromatic residues sits between two BhFhbA residues: Phe154 from the hinge region and Tyr170 from helix α8. This tightly constrained binding pocket is further coordinated by the hydrophobic sidechains of α6 Met137 and Leu146 and α8 Ile171. Moreover, towards the C-terminus of FH20, Tyr199 from BhFhbA α9 forms van der Waals interactions with FH20 Val1200, with the closest approach being 3.8 Å (Fig 1B).

There are two crystal structures of microbial proteins complexed with FH19-20: OspE from *Bo*. *Burgdorferi* [22] and BhFhbA. We compared the binding mechanisms between the two proteins. Previous binding inhibition assays using 15 mutants of FH20 showed that the binding-sites of BhFhbA and OspE overlap [30]^,^ and that BhFhbA inhibited binding of OspE to FH19-20 and *vice versa*. We superimposed the FH19-20s of the two complex structures to analyze the similarities and differences in the binding. We identified five key amino acids in FH20 that form hydrogen bonds to both OspE and BhFhbA (S1 Table and S2A Fig). Thus, BhFhbA and OspE have completely unrelated folds, but nevertheless utilize the same surface and partially similar contacts to bind FH20 (S2A Fig). Comparison to the structure of FH19-20 in complex with sialic acid and C3d (PDBID: 4ONT [40]) revealed that the same surface on FH20 is also occupied by sialic acid and, moreover, that FH20 W1183 makes similar interactions with the sialic acid moiety as with the F154 of FhbA (S2B Fig). This supports our conclusion that the primary interface seen in the crystal structure is the biologically relevant one.

### Mutagenesis and binding assays reveal important amino acids in the hydrophobic binding pocket

**Fig 2:**
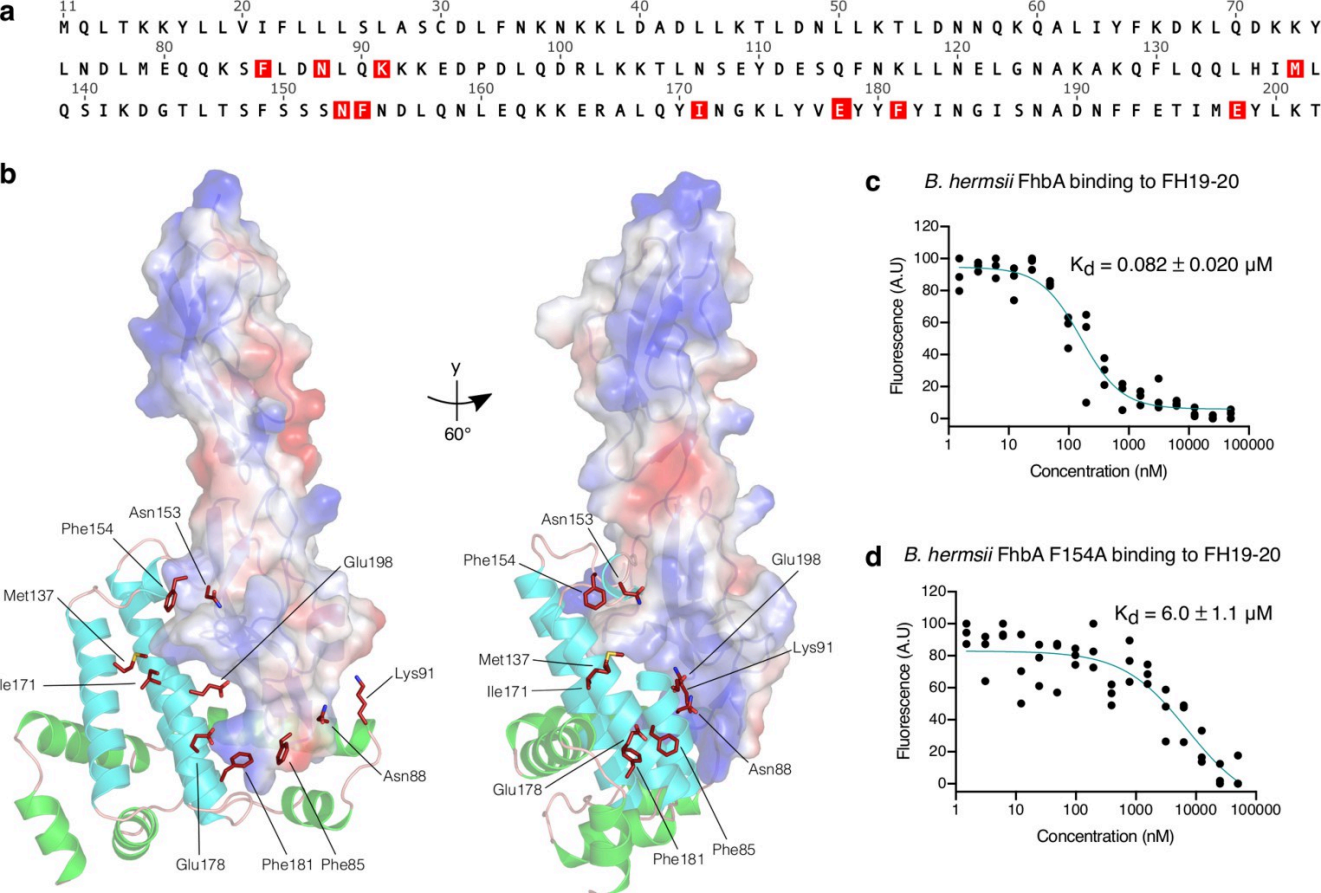
Binding of FH20 to BhFhbA is mediated by a hydrophobic binding pocket. (a) The sequence of BhFhbA from *Borrelia hermsii* YOR (UniprotID: W5SB08) with mutated residues marked in red. (b) Positions of the mutations mapped on the structure of the BhFhbA:FH19-20 complex. BhFhbA α-helices 1–5 are shown in green, 6–9 in teal (cartoon), and FH19-20 is shown as an electrostatic surface model. (c) Binding of FH19-20 to wild type BhFhbA and (d) Phe154Ala mutant BhFhbA detected by MST. *K*_d_-values ± SEM (μM) calculated from three technical replicates (normalized fluorescence values) and individual data points are shown.

Next, we designed ten alanine point mutations located on the interface of BhFhbA that binds FH19-20. A group of mutations (Asn153Ala, Phe154Ala, Met137Ala, Ile171Ala) targeted at the hydrophobic binding pocket, whereas the rest (Phe85Ala, Asn88Ala, Lys91Ala, Glu178Ala, Phe181Ala, Glu198Ala) were targeted to the interface below the hydrophobic cavity (Fig 2A and 2B). The single alanine mutants of BhFhbA were expressed in *E*. *coli* as 6x-His fusion proteins and purified using Ni-NTA and size-exclusion chromatography.

We used affinity ligand binding immunoblot as an initial robust screen for the effects of mutations on the binding to FH19-20 (S3 Fig). Some mutations decreased binding, and one (Phe154Ala) completely abolished it. Next, we used fluorescence-based MicroScale Thermophoresis (MST) to determine the binding affinities of the BhFhbA mutants to FH19-20 (Table 2 and S4 Fig). MST measurements showed ~70-fold decrease in binding of the Phe154Ala mutant (*K*_d_ = 6.0 μM) to FH19-20 compared to the wild type protein (*K*_d_ = 0.082 μM) (Fig 2C and 2D). This was also confirmed by gel filtration chromatography, where no complex formed between the Phe154Ala mutant and FH19-20 (S5 Fig). In addition, we compared the binding of wild type BhFhbA and the Phe154Ala mutant to full length FH using MST. As with FH19-20, wild type FhbA bound FH with high affinity (*K*_d_ = 30 nM) and Phe154Ala mutant showed no binding (S6A Fig).

**Table 2:** Binding affinities of BhFhbA and mutants measured by MST. Mean binding affinities (*K*_d_ in μM concentration ± SEM) calculated from three individual experiments (S4 Fig). Fold decrease in binding was calculated by normalizing the *K*_d_ values relative to wild type BhFhbA (assigned a value of 1). *Designates *p*-value < 0.05 from an unpaired *t*-test in comparison to the wild type. n = 3 for each protein.

Mutant number	Mutation	*K*_d_ (μM)	Fold decrease in binding
	Wild type BhFhbA	0.082 ± 0.020	1
#1	Phe85Ala	0.11 ±0.025	1.34
#2	Asn88Ala	0.11 ± 0.043	1.34
#3	Lys91Ala	0.11 ± 0.025	1.34
#4	Met137Ala	1.0 ± 0.23*	12.2
#5	Asn153Ala	0.073 ± 0.024	0.890
#6	Phe154Ala	6.0 ± 1.1*	73.2
#7	Ile171Ala	0.38 ± 0.12	4.63
#8	Glu178Ala	0.10 ± 0.031	1.22
#9	Phe181Ala	0.033 ± 0.0090	0.402
#10	Glu198Ala	0.13 ± 0.020	1.59

In the binding data, we observed a 12-fold and a 5-fold decrease in the affinities of two other mutants, Met137Ala and Ile171Ala for FH19-20, respectively (Table 2). In the complex, Trp1183 from FH20 is elegantly slide in between the coordinated aromatic binding stack of four hydrophobic residues Met137, Leu146, Phe154, and Ile171 (Fig 1B), providing a structural explanation for these biochemical results. However, most single alanine substitutions of charged residues had no effect on binding, suggesting that hydrophobic interactions dominate. To confirm the dominance of hydrophobic interactions further, we compared complex formation in high-salt conditions (PBS + 500 mM NaCl) and physiological environment (PBS) by gel filtration (S5 Fig). High ionic strength did not disrupt the binding of BhFhbA to FH19-20, supporting our proposal that the interaction is mainly hydrophobic, although involvement of other charged residues in the interaction cannot be excluded.

To exclude the possibility that the critical effect of the Phe154Ala mutation on binding to FH is due to protein misfolding, we performed CD-spectroscopy and demonstrated that wild type and Phe154Ala mutant proteins have identical secondary structure profiles, indicating that both are correctly folded (S7 Fig).

### FhbA interacts with FH mainly *via* binding to FH20

*B*. *hermsii* was originally reported to acquire complement regulator FHL-1 from serum, as well as to bind a cloned fragment of FH containing domains 1–7 [31]. Domains 6–7 are identical in FH and FHL-1 and contain a binding site for several microbial proteins, like fHbp of *Neisseria*, CspA of Lyme disease borrelia and streptococcal M-protein [41]. We therefore decided to test whether BhFhbA binds this region by using an FH fragment of domains 5–7 (FH5-7).

**Fig 3:**
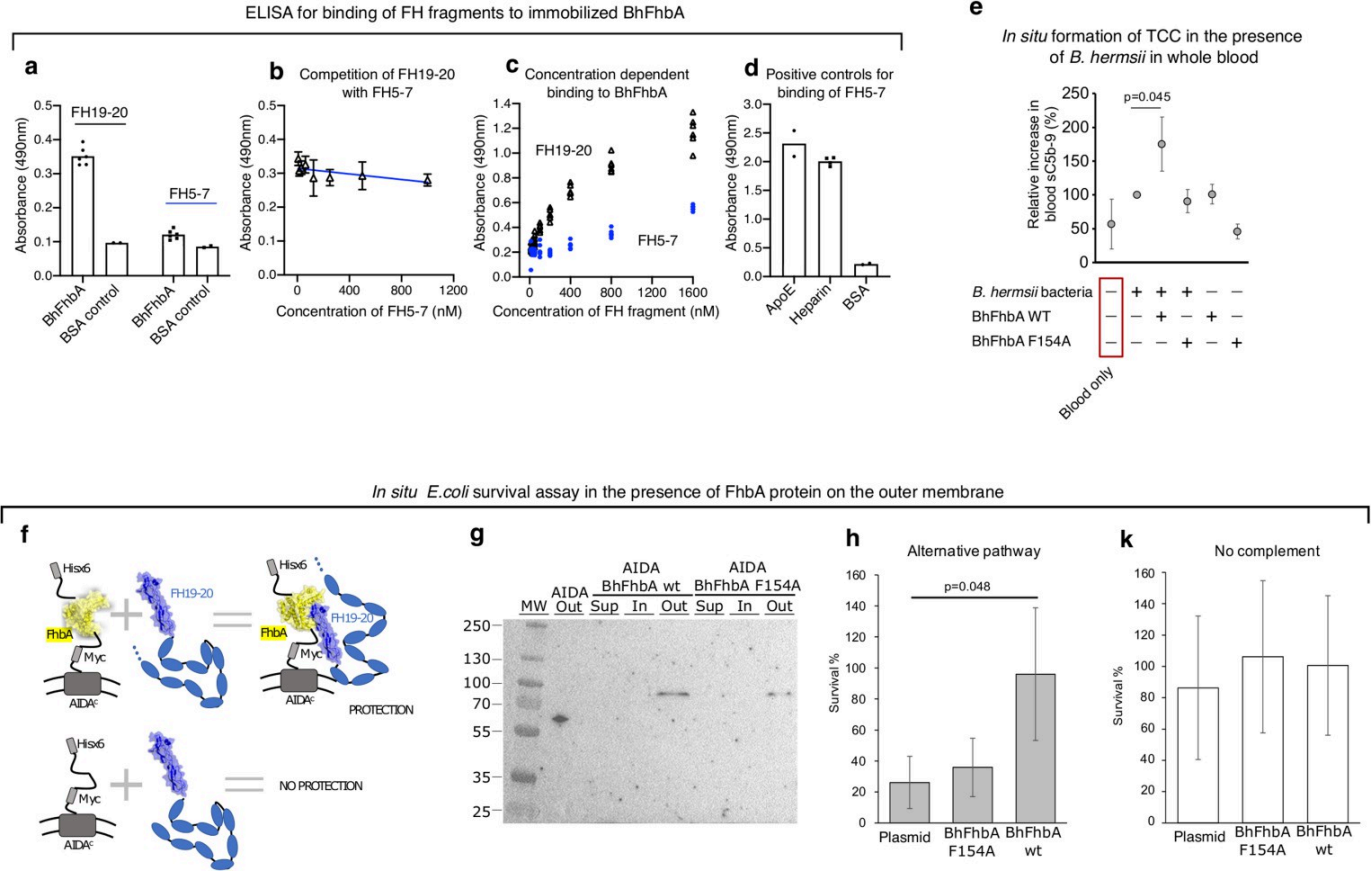
BhFbA affects complement activation and survival. (a) Binding of FH19-20 and FH5-7 to immobilized BhFhbA detected by ELISA. Individual data points are shown with bars indicating mean values. Values shown in panels *a-e* are absorbances measured by ELISA. (b) Effect of FH5-7 on binding of FH19-20 to immobilized BhFhbA detected by ELISA. n = 5 or more. Error bars represent S.D. (c) Concentration-dependent binding of FH19-20 and FH5-7 to BhFhbA. Individual data points from several ELISA assays are shown. (d) Controls for FH5-7 fragment confirmed by ELISA. Binding to ApoE [65] protein and heparin were used as positive controls, and BSA as negative control. Individual data points from assays are shown, and bars indicate mean values. (e) Complement activation measured by formation of soluble terminal complement complexes (TCC) in whole blood. Proteins added to reactions are shown below the graph. Data are presented as relative TCC-amount (%) compared to the sample where bacteria alone were incubated in blood (n = 4, error bars represent S.D.) Difference between bacteria alone to bacteria incubated with wild type FhbA (* p<0.05) calculated by paired *t*-test (Mann-Whitney U-test for independent samples). (f) Schematic representation of the survival assay using the AIDA-1 transport system^44^, where binding of membrane expressed FhbA to serum FH protects *E*. *coli* against complement attack. (g) Western blot showing the presence of His-tagged BhFhbA on the outer surface of *E*. *coli*. Outer membrane sample of *E*. *coli* expressing an empty AIDA1 system shows a band present at about 63 kDa, consistent with the expected molecular weight (lane 2). BhFhbA wt and Fhe154Ala mutant proteins are present only in outer membrane (out) at the expected molecular weight of 83 kDa (lanes 5 and 8). The anti-His signal was not detected in the supernatant fraction (sup) or in the inner membrane (in). (h) Serum survival of *E*. *coli* clones expressing BhFhbA, BhFhbA mutant F154A and empty vector (AIDA) as a control. Result was calculated as a percentage of bacteria that survived after 15 min incubation in serum as compared to the number of bacteria at time point zero (n = 4, error bars represent S.D.) Difference between BhFhbA clone to control significant (* p<0.05) calculated by one-way ANOVA supplemented with Dunnet’s test for unequal variances. (i) Survival of *E*. *coli* clones expressing BhFhbA, BhFhbA mutant F154A and empty vector as a control in the presence of inactivated (with 10 mM EDTA) serum without complement. Result was calculated as a percentage of colonies surviving after 15 min incubation in media as compared to the number of bacteria at time point zero (n = 3, error bars represent S.D.).

BhFhbA was immobilized to ELISA-plates, and purified FH5-7 or FH19-20 fragments were added. After washing steps, the binding of FH5-7 and FH19-20 was detected by a polyclonal anti-FH antibody. When compared to FH19-20, the FH5-7 fragment displayed very modest binding to BhFhbA (Fig 3A–3D). Moreover, FH5-7 did not affect the binding of FH19-20 to BhFhbA in a competition assay (Fig 3B). To analyze the binding interaction in another setup, we used MST to test if FH5-7 or FH19-20 affects the binding of full-length FH to BhFhbA in a competition assay. FH5-7 slightly decreased the binding of full-length FH to BhFhbA, but FH19-20 abolished it completely (S6B Fig).

These results suggest that BhFhbA binds FH predominantly *via* domain 20. We cannot exclude that under some circumstances, BhFhbA also interacts with FHL-1 or FH *via* domains other than 20, but the major binding site for BhFhbA is clearly FH domain 20.

### FhbA decreases complement activation and enhances serum survival

After entering the body, RF *Borrelia* can survive and multiply in the blood, and cause massive spirochetemia, where bacteria are present in the blood at high densities (10^5^−10^6^ bacteria/ml). We mimicked natural conditions by incubating 50,000 live *B*. *hermsii* bacteria in 100 μl of whole blood treated with hirudin to prevent coagulation, and measured the amount of terminal complement complexes (TCC) as indicators of complement activation [42]. We first confirmed that, as expected, the presence of bacteria in blood increases complement activation, which is seen as an increase in the amount of TCC in the sample. Adding purified BhFhbA to the reaction to inhibit binding of FH to *B*. *hermsii* led to even higher levels of TCC compared to bacteria alone. Conversely, adding of the FH-binding defective mutant BhFhbA/Phe154Ala had no effect on the levels of TCC, which were similar to bacteria alone (Fig 3E). Some enhancement of complement activation was also detected when BhFhbA was incubated in the absence of bacteria, whereas BhFhbA/Phe154Ala had no effect. We hypothesize that high-affinity binding of BhFhbA to FH may affect its ability to regulate complement in the fluid phase and/or can lead to formation of complement activating immune complexes.

We then expressed BhFhbA wild type and BhFhbA/Phe154Ala mutant on the outer membrane of *E*. *coli* to study how binding of FH affects bacterial survival. We chose *E*. *coli* as it is a Gram-negative bacterium, and laboratory strains lacking any evasion mechanisms are efficiently killed by complement [43]. We utilized the autotransporter adhesin involved in diffuse adherence-I (AIDA-I) system [44] (Fig 3F) to deliver the BhFhbA protein to the outer surface of *E*. *coli* and showed by immunoblot analysis that His-tagged BhFhbA was present in the outer membrane fraction (Fig 3G). We then performed serum sensitivity assays by utilizing BhFhbA wild type and mutant proteins (see Materials and Methods). Survival of the strain expressing wild-type BhFhbA was significantly (*p*<0.05) higher when compared to the control strain and to the strain expressing BhFhbA/Phe154Ala mutant (Fig 3H). However, in the absence of complement, all three strains showed similar survival (Fig 3I). These results show that binding of functional BhFhbA to FH is necessary and sufficient for increasing the survival of the bacteria.

### A new family of immune evasion proteins revealed by bioinformatic searches

The crystal structure of BhFhbA inspired us to study the distribution of FhbA-like and other FH-binding proteins within the whole *Borreliaceae* family. First, we performed a thorough search for homologous proteins within all available whole genomes (in total 154) and separately deposited sequences from the family *Borreliaceae* (Table 3). Analysing sequence data from borrelia is demanding, as *Borreliaceae* have very complex genomes with both linear and circular plasmids [45] where length, diversity and composition vary between different species. It is thus possible that some FH-binding proteins might be absent from the databases due to plasmid loss, which has been reported in the *Borreliella* clade.

**Table 3:** *fhbA*-related, *cspA* (CRASP-1/BBA68), *cspZ* (CRASP-2) and *ospE* genes reported in *borreliae*. ‘+’ means that the gene has been identified at least in one source.’–‘means that the sequence was not found in any of the sources used. Data were acquired from the sequenced genomes, published reports and individual sequences deposited to the databases. Relevant genes were searched as described in Materials and Methods. Accession numbers for FhbA-related proteins are listed in the legend to Fig 4.

Clade	Species	Genes			
		*fhbA*	*cspA* (CRASP-1)	*cspZ* (CRASP-2)	*ospE (CRASP-5)*
Lyme disease (LD) clade	*Borreliella burgdorferi*	-	+	+	+
	*Borreliella finlandensis*	-	+	-	+
	*Borreliella bissettiae*	+^**1**^	+	+	+
	*Borreliella mayonii*	-	+	-	+
	*Borreliella spielmanii*	-	+	+	+
	*Borreliella afzelii*	+^**2**^	+	+^3^	+
	*Borreliella japonica*	-	+	-	+
	*Borreliella garinii*	-	+	+	+
	*Borreliella bavariensis*	-	+	-	?^**4**^
	*Borreliella valaisiana*	+^**5**^	+	+	+
	*Borreliella chilensis* ^**5**^	-	-	-	-
Relapsing fever (RF) clade	*Borrelia parkeri*	+	-	-	-
	*Borrelia turicatae*	+^**6**^	-	-	-
	*Borrelia coriaceae*	+	-	-	-
	*Borrelia hermsii*	+	-	-	-
	*Borrelia anserina* ^**7**^	-	-	-	-
	*Borrelia duttonii*	+	-	-	-
	*Borrelia recurrentis*	+	-	-	-
	*Borrelia crocidurae*	+	-	-	-
	*Borrelia hispanica*	+	-	-	-
	*Borrelia persica*	+	-	-	-
	*Borrelia miyamotoi*	+	-	-	-

^1^ *fhbA*-related gene is detectable at the DNA level, but appears truncated if translated *in silico*

^2^ *fhbA*-related protein is not conserved in the FH-binding position Phe_154_

^3^ *cspZ* described in [46]

^4^*ospE* has 30% identity to the N-terminal half of another hypothetical protein BGP333 (GenBankID:AAU86184): the FhbA-related protein is truncated

^5^ Strain isolated from ticks [47]

^6^ FhbA-related protein is not found in the genome available, but protein has been detected^32^

^7^ Causative agent of avian spirochetosis [48]

**Fig 4:**
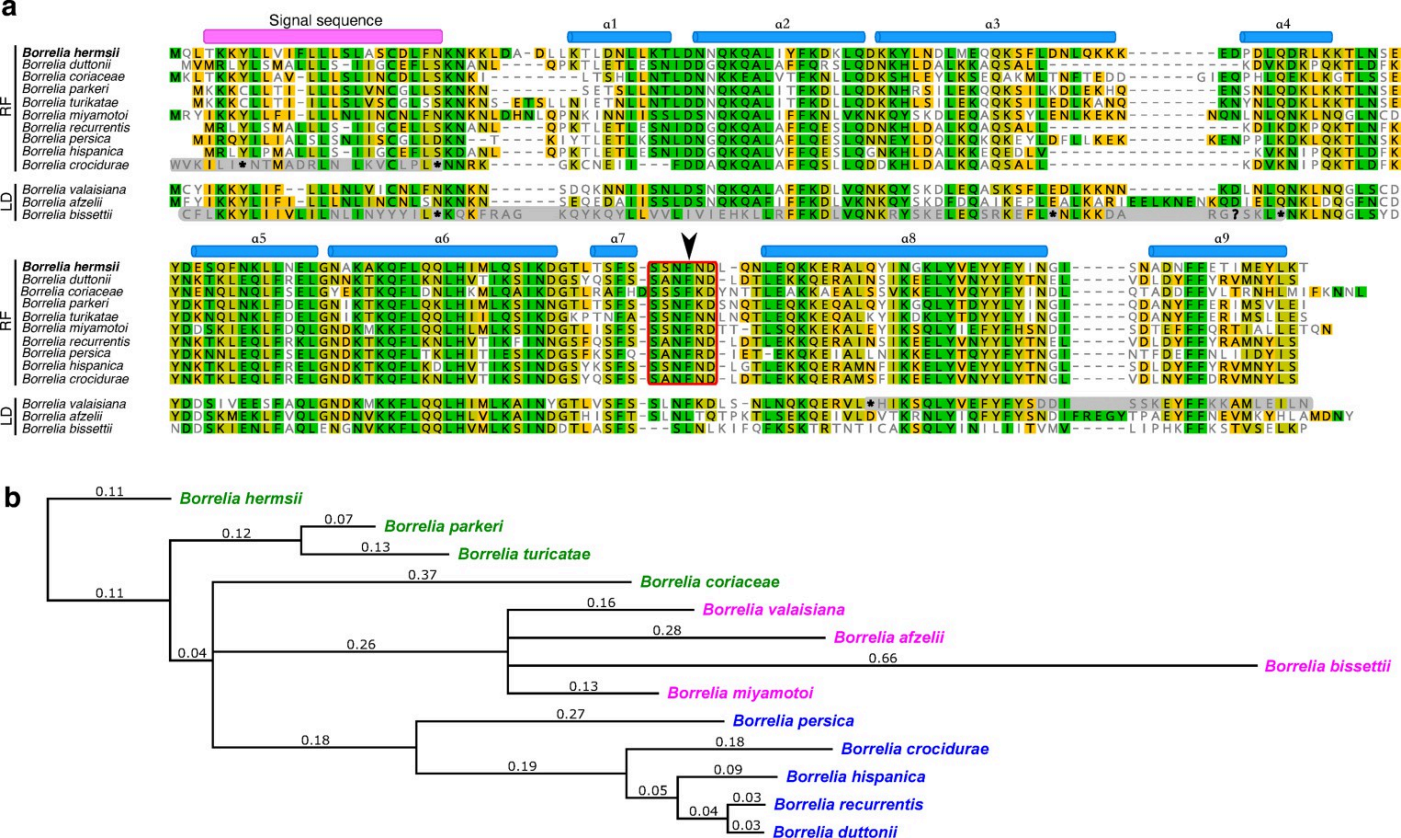
A new family of immune evasion proteins in borreliae revealed by bioinformatics searches. (a) Multiple sequence alignment of the FhbA-related proteins. The signal sequence is marked in magenta and secondary structure elements derived from the crystal structure of BhFhbA:FH19-20 are shown in blue above the sequence. The conserved hinge region is marked with a red rectangle, and an arrowhead points at the key Phe residue important for tight binding to FH20. The sequence alignment consists of protein sequences, predicted protein sequences and translated genomic regions that match the BhFhbA used as the search sequence (see Materials and Methods for details). Asterisk ‘*’ represents a stop codon and question mark ‘?’ stands for an incomplete codon, where a frameshift appears to have occurred. In generating the protein alignment, the frameshift was ignored, and the translation frame was preserved to allow further protein alignment after that problematic codon. Sequence parts with grey background are translated genomic sequences, which most probably are not present in derived proteins due to stop codons or frameshifts. Accession numbers and references for the sequences used in the alignment: *B*. *hermsii* YOR (W5SB08), *B*. *coriaceae* Co53 (W5T1N6), *B*. *duttonii* CR2A (W6TXL9), *B*. *parkeri* (D5GU46), *B*. *turicatae* (B0L8C8), *B*. *miyamotoi* (A0A075BUA1), *B*. *recurrentis* (C1L349), *B*. *persica* No12 Bp4780 (contig: NZ_AYOT01000066.1, nucleotides: 4020 -> 4611), *B*. *hispanica* CRI Bhis_2727 (contig: NZ_AYOU01000105.1, nucleotides: 3668 -> 4235), *B*. *crocidurae* str. Achema (contig: NC_017778.1, nucleotides: 46553 -> 45974), *B*. *valaisiana* VS116 (C0R979), *Bo*. *afzelii* (WP_011703930.1), *B*. *bissettiae* DN127 (contig: NC_015916.1, nucleotides: 3284 -> 2685). (b) Phylogenetic tree of the FhbA-family proteins constructed from the sequences shown in (a). The numbers represent the substitutions per position and the length of the lines is equivalent to these numbers. The three colours emphasize that the sequences cluster into three distinct groups.

Our structure-guided sequence database analysis approach allowed us to reliably identify 10 species with sequences homologous to BhFhbA in RF borreliae clade, and 3 species in the LD group (Figs 4A and S8). A phylogenetic tree of these homologous proteins (Fig 4B) shows that they form three different clusters. Nonetheless, in all 10 species of RF *borreliae*, the residue corresponding to Phe154, which is essential for FH-binding in BhFhbA, is conserved. In *B*. *crocidurae*, both sequenced strains (*Achema* and *DOU*) have stop codons in the signal sequence that alter the amino terminal regions of the proteins. It would be interesting to see if these truncated genes translate into functional proteins and provide protection like FhbA protein from *B*. *hermsii*.

The translated genomic regions of three species from the LD borreliae clade aligned well with other BhFhbA proteins, although with some changes (Fig 4A). In *Bo*. *valaisiana*, the protein is two helices shorter, but the hinge region and the key Phe154 residue mediating FH-binding are conserved. *Bo*. *bissettiae* (NC_015916.1) has a Leu instead of conserved key Phe, several stop codons, and a single nucleotide deletion that leads to a frame shift (marked by ‘?’ in Fig 4A). The only sequence that seems not to be altered at the DNA level is from *Bo*. *afzelii*. Potentially it can be translated into functional protein, though it has Leu instead of conserved Phe, as in *Bo*. *bissettiae*. Further studies are required to test whether any of these genes are expressed and provide similar protection as FhbA.

Overall, it appears that FhbA-like genes are present in all RF clade species, except for *B*. *anserina*, and that the key binding loop containing Phe154 is conserved. However, although FhbA-like genes are identifiable at the DNA level also in the LD clade as well, it is not known if they encode functional proteins.

### Identification of a conserved binding mechanism for FH binding

**Fig 5:**
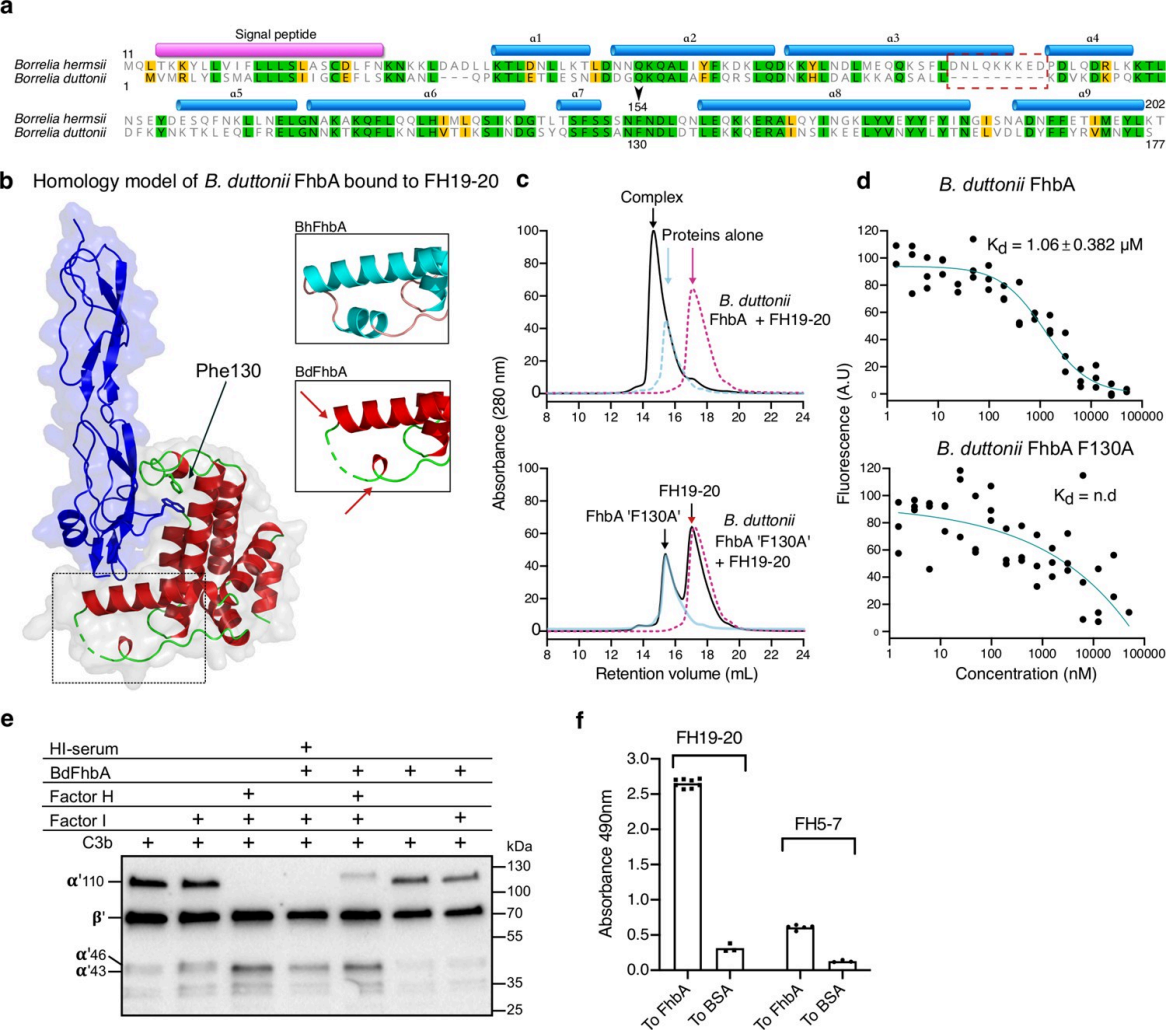
FhbA-related protein from *B*. *duttonii* (BdFhbA) utilizes the same binding mechanism to FH20 as BhFhbA. (a) Sequence alignment of FhbA proteins from *B*. *duttonii* and *B*. *hermsii* shows a difference in the region between helices 3 and 4 (dashed red rectangle). (b) Structure-based homology model of BdFhbA shows almost identical overall structure to BhFhbA except shortening of helices 3 and 4. (c) Elution profiles of the proteins alone and in combination with each other on the size exclusion column. FH19-20 protein elution profile in magenta, wild type and Phe130A variant BdFhbA in light blue, and a combination of the two proteins in black curves. In each chromatography run, 100 μl of sample containing 20 nmole of each of the tested protein(s) was injected. (d) Binding of FH19-20 to BdFhbA and BdFhbA/Phe130A variant as detected by MST. *K*_d_-values with SEM (μM) calculated from three technical replicates (normalized fluorescence values) are shown for the wild type BdFhbA. For the BdFhbA/Phe130A variant, the MST software could not reliably determine a value for *K*_d_. (e) Cofactor activity of FH in factor I mediated cleavage of C3b. FH from serum or after purification was bound to BdFhbA coated on microwell plate. After washing, C3b and/or Factor I were added. C3b and its cleavage fragments (α’46 and α’43) were detected using anti-C3d antibody. (f) Binding of FH19-20 and FH5-7 to surface coated BdFhbA. Individual data points from ELISA are shown, and the bars represent mean values.

To examine if the BhFhbA binding mechanism we identified occurs in other members of this protein family, we cloned, expressed and purified a novel FhbA homologue from *B*. *duttonii* (BdFhbA). We chose this protein, because *B*. *duttonii* is known to bind FH [28]. BdFhbA protein has high conservation (41.8% identity) to BhFhbA (Fig 5A). We modelled the structure of BdFhbA with Phyre2 using our crystallized BhFhbA as a template, and observed an almost identical structure, except for shorter helix 3 and its connection to helix 4, as expected from the sequence alignment (Fig 5A and 5B). The location of the helices, the positioning of the hinge area, and the orientation of the critical residue Phe130 (corresponding to BhFhbA Phe154 in *B*. *hermsii*) were conserved.

We next measured binding of FH19-20 to BdFhbA. Gel filtration experiment showed that the proteins form a stable complex (Figs 5C and S9). Moreover, MST experiments revealed that FH19-20 bound BdFhbA (Fig 5D), although with lower affinity (1.06 ± 0.38 μM) than to BhFhbA (Fig 2 and Table 2). The lower affinity might be due to small differences in the hydrophobic binding pocket. For example, BhFhbA Met137, which sits in the hydrophobic cavity, is replaced by Thr in BdFhbA. We also mutated the key phenylalanine (corresponding to Phe154 in BhFhbA) in BdFhbA to alanine (Phe130Ala). As expected, both the gel filtration (Figs 5C and S9) and MST assays (Fig 5D) showed a drastic decrease in the binding of FH19-20 to BdFhbA/Phe130Ala. We also showed that full length FH binds wild type BdFhbA but not the BdFhbA/Phe130Ala mutant (S10 Fig) and retains its cofactor activity in FI-mediated cleavage of C3b (Fig 5E). Finally, BdFhbA binds FH predominantly *via* domain 20 and possesses only weak affinity towards FH5-7 (Fig 5F), like BhFhbA. CD spectrometry showed that both wild-type BdFhbA and BdFhbA/Phe130Ala were correctly folded (S7 Fig).

Together, these results clearly show that two homologous proteins, BhFhbA and BdFhbA, bind FH19-20 *via* a novel, conserved mechanism. Based on structure-assisted multiple-sequence alignments, we predict that other members of the family utilize the same mechanism.

### Delineation of FH-binding protein families of *Borrelia*

The most extensively studied species from the LD clade, *Bo*. *burgdorferi*, has five different FH-binding proteins: CspA/BBA68/BbCRASP-1, CspZ/BBH06/CRASP-2, ErpP/BBN38/OspE/CRASP-3, ErpC/CRASP-4, ErpA/BBP38/BBL39/OspE/CRASP-5, with the last three being highly homologous to each other [27]. Below we use gene name as the protein name, as it is the accepted practice for microbial proteins, along with the well-known general name (complement regulatory acquiring surface protein: CRASP).

We delineated the full spectrum of potential FH-binding proteins in LD and RF clades using the same set of genomes as for the FhbA searches to identify homologues for FH-binding proteins from *Bo*. *burgdorferi*. We used sequences from the known structures of CspA/CRASP-1[49], CspZ/CRASP-2[50] and OspE [22] in the search. Ten of the eleven genomes analyzed in the LD clade have proteins homologues to CspA/CRASP-1, six of eleven to CspZ/CRASP-2 and ten of eleven to OspE/CRASP-5 (in *Bo*. *bavariensis* the protein has an N-terminal region displaying 30% identity to OspE (Tables 3 and S3). In contrast, the members of the RF clade have neither CspA/CRASP-1 nor CspZ/CRASP-2 homologues.

There were two species that lacked all four classes of FH-binding proteins (Table 3). The first is *Bo*. *chilensis*, which was originally isolated from ticks [47], and currently there is no data about its vertebrate host. The second is *B*. *anserina*, a bird isolate. Although another bird infecting species, *Bo*. *garinii*, was shown to bind avian FH [51], it is not known whether *Bo*. *anserina* binds FH at all.

Taken together, FH binding proteins clearly fall into four groups, which also coincide with the phylogenetic classification of the borreliae species. All LD clade species with FH-binding proteins have CspA/CRASP-1 proteins, and the majority also possess CspZ/CRASP-2 and OspE/CRASP-5 -proteins. RF clade species exclusively have FhbA proteins and lack the other three, suggesting a significant role for FhbA proteins in complement evasion of RF borreliae.

## Discussion

By determining a 2.2 Å resolution crystal structure of *B*. *hermsii* surface protein BhFhbA in complex with FH19-20, we revealed the molecular mechanism by these two proteins interact with each other. The structure, combined with mutagenesis and binding studies, led to the identification of a conserved aromatic residue, Phe154, that is central in binding FH. Using structure-guided sequence analysis with the structure of BhFhbA as a search model, we identified several putative homologous proteins in relapsing fever and Lyme disease borreliae. To confirm the common binding mechanism between the FhbA-related proteins and FH, we also expressed a novel FH19-20 binding protein (BdFhbA) from *B*. *duttonii*, which causes relapsing fever, and mutated the key residue, Phe130. MST and gel filtration demonstrated that these two proteins share the same FH-binding mechanism. We also delineated four different FH-binding proteins families of *Borrelia/Borreliella* and show that the LD clade of borreliae has four different groups of FH-binding proteins, but the RF borreliae clade appears to possess only one.

BhFhbA was originally identified as an FH binding protein from *B*. *hermsii*, which causes relapsing fever [29]. The protein was predicted to be composed of four α-helices flanked by three loops. When compared to our crystal structure, the locations of predicted coiled coils and loop regions match poorly. Thus, earlier random [31] and site-directed mutagenesis [52] studies aimed at the predicted loop regions of the protein also targeted α-helical and core regions of the protein, affecting secondary structure elements and protein folding. We mapped six previously published mutants with reduced or no FH-binding activity [31] to our structure (S11 Fig). Detailed inspection of the environment of each mutated position explains decreased binding. For example, the Asn172Thr mutation disrupts two hydrogen bonds that keep helices 2 and 3 together. Though Asn172 is located far from the active hinge region, such a mutation is likely to affect overall folding or stability of the protein. Nevertheless, these earlier studies support the importance of the hinge region in FH binding.

BhFhbA binds to domain 20 of FH (Figs 1A and S1). Structural analysis of FH19-20 complexes with two microbial proteins, FhbA and OspE from *Bo*. *Burgdorferi* [22] suggests that the general microbial binding site on FH20 mediates interaction (S1 Table). However, the interactions are different: a hydrophobic binding pocket is formed between FH20 and BhFhbA, whereas the FH20:OspE interaction is mainly electrostatic in nature and mediated by hydrogen bonds. Binding of FH to sialic acid on erythrocytes, endothelial cells and platelets has been shown to protect host cells from complement [53]. Interestingly, the structure of the FH19-20:sialic acid:C3d [40] complex revealed that the same general microbial binding site in FH20 is involved in binding to sialic acid. This is a rare and interesting example of convergent evolution of a binding site utilization; the binding ligands and mechanisms are different, but the binding patch site on FH largely overlaps.

The other important interaction site in FH is in domains 6–7, part of which is present in full-length FH as well as in FHL-1. Typically, microbes bind FH *via* domain 20, or FH and FHL-1 *via* domain 7. BhFhbA is a rare example, as it was first reported to bind FHL-1 and FH fragment 1–7 [54] and later FH *via* domain 20 [30]. Here, we examined if BhFhbA has two binding sites on FH by comparing the interactions of FH19-20 and FH5-7 to FhbA (Figs 3A–3D and S6). Our results suggest that FhbA has only weak affinity for FH5-7 and it cannot compete with FH19-20 in binding to BhFhbA (Figs 3B and S6). When all FhbA-related proteins analyzed so far are considered, this result is not surprising. No binding of FH1-7 to FhbA-related protein HcpA from *B*. *recurrentis* [33], to BpcA of *B*. *parkeri* [32] or to CbiA of *B*. *miyamotoi* [40] was observed. It cannot, however, be excluded that under certain circumstances, e.g., in specific tissue locations, RF borrelia acquire FHL-1 as well, but the most important binding site for this protein is on FH20.

**Fig 6:**
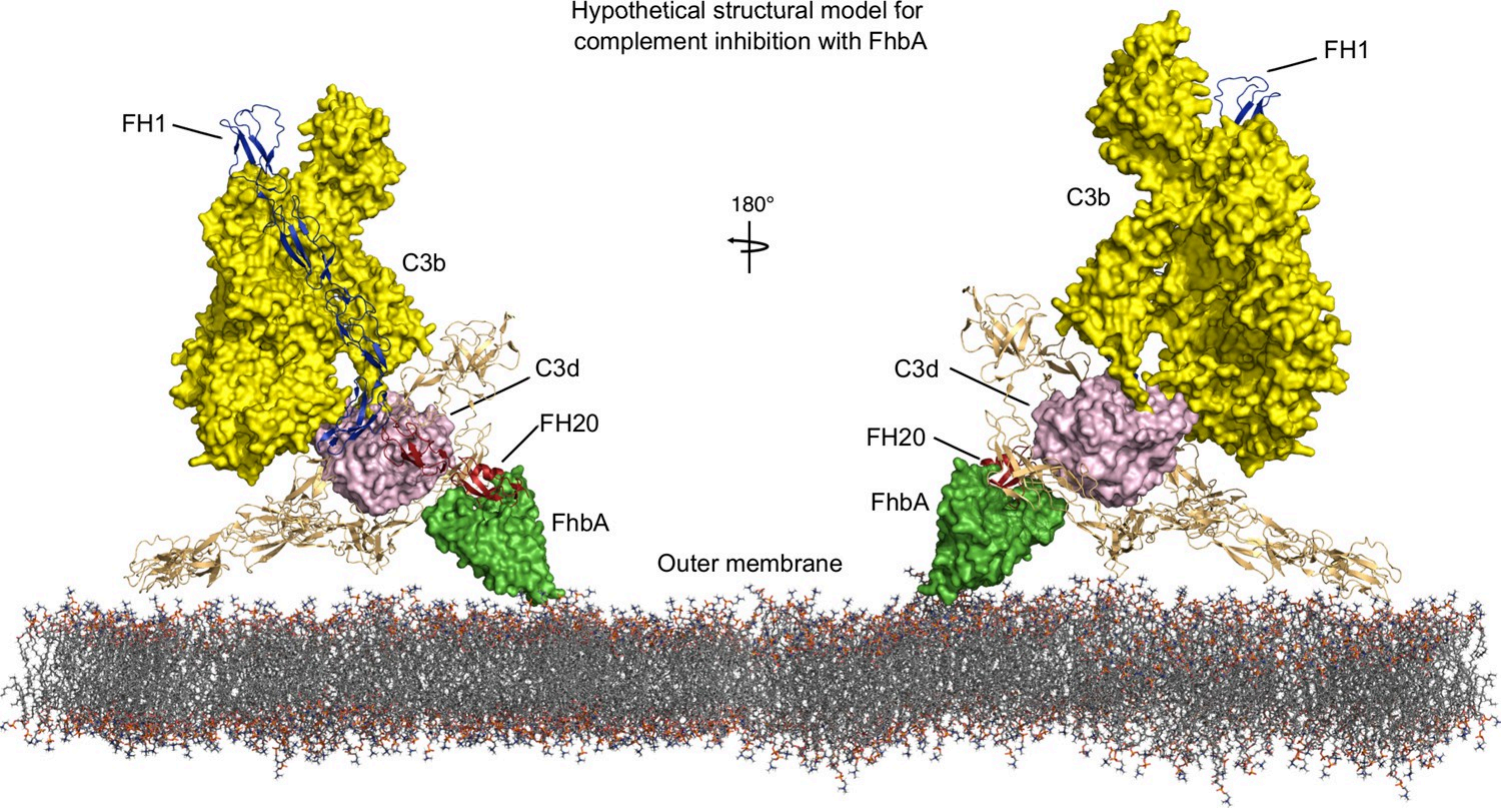
A working model for how BhFhbA recruits FH to mediate immune evasion. Membrane-bound BhFhbA (bright green) recruits FH (cartoon representation) of the host through binding site on domain 20 of FH (in red). When bound to BhFhbA, FH domains 1–4 are free to bind to C3b (yellow) and inhibit complement. When bound to microbial protein *via* domain 20, FH can also bind C3d fragment (in pink) *via* domain 19. Full-length FH model was manually constructed in Pymol by combining existing structures of different subcomplexes. Model for FH1-4 bound to C3b was from PDB2wii [15]. Structures of FH19-20 (in red) bound to C3d were obtained from PDB entries 5nbq [56] and 2xqw [66].

Microbes bind domains other than N-terminal domains FH1-4 so that this region can bind to and downregulate C3b. It has been previously shown that FH bound to BhFhbA retains its cofactor-activity in cleaving C3b both using purified proteins [30] and when BhFhbA is expressed on the bacterial cell surface [55]. We confirmed that FH bound to BdFhbA retains its cofactor-activity (Fig 5E). Furthermore, we previously showed that FH20 bound to BhFhbA or other microbial proteins enhances the cofactor-activity of FH in cleaving C3b [30]. The mechanism of this enhanced regulatory function is not clear, but we speculate that simultaneous binding to target *via* domain 20 and to C3b *via* domain 19 facilitates enhanced activity. Indeed, we previously solved the structure of a tripartite complex between microbial protein OspE, C3d and FH19-20 [56]. Our model of FhbA on the surface of *Borrelia* is based on the hypothesis that BhFhbA acts similarly to OspE and binds simultaneously to FH20 and C3d (Fig 6).

Using BhFhbA as a search model in structure-guided sequence database analysis enabled us to identify 10 homologous DNA loci from the RF and three from the LD clades (Fig 3). All ten identified proteins in the RF borreliae group are very similar within the FH20 binding region, as both the key phenylalanine residue and the surrounding hinge region are highly conserved. Moreover, for BhFhbA and BdFhbA, mutations of the key phenylalanine dramatically affected binding to both FH19-20 and full-length FH (Figs 2, 4, 5, S4, S6 and S10). The only exception is *B*. *turicatae*, which has an asparagine instead of aspartic acid in the +2 position after Phe154. Consistent with our predicted binding mechanism, *B*. *turicatae* BtcA is the only FhbA-related protein of RF borreliaea that does not bind FH [32] (Table 4). Interestingly, there are three FhbA-related sequences in the borrelia from the LD clade, which cluster separately from the RF group (Fig 4B). Sequence data show that there are many deleterious alternations at the DNA level. It is not yet known, if these FhbA-related sequences are functional on a protein level.

**Table 4:** Suggested interaction partners for the FhbA-related proteins.

Species/ strain	Protein	Uniprot accession	FH	FH20	FHL1	C3	C3b	C4	C4b	C5	Pl
*B*. *hermsii/* YOR	BhFhbA [54]	W5SB08	+	+	+	n.s.	n.s.	n.s.	n.s.	n.s.	+
*B*. *hermsii*/ HS1	BhFhbA [59]	A1KEE1	+	+	-	n.s.	n.s.	n.s.	n.s.	n.s.	+
*B*. *parkeri*/ RML	BpcA [32]	D5GU46	+	+	-	-	-	-	-	-	+
*B*. *miyamotoi*/ HT31	CbiA [34]	A0A075BUA1	+	+	-	+	+/-	-	+	+	n.s.
*B*. *recurrentis*/ AI	HcpA [33]	C1L349	+	+	-	+	+	-	+	-	+
*B*. *turicatae*/ 91E135	BtcA [32]	B0L8C8	-	-	-	-	-	-	-	+	+

+ = has been shown to bind/interact, +/- = weak/uncertain interaction,— = no binding/interaction, n.s. = not studied, Pl = plasminogen

Analysis of all available sequence data from borreliae demonstrates that the LD clade has evolved to have three to four different classes of FH-binding proteins, whereas the RF clade has just one, which we name the FhbA-related protein family (Table 4). FhbA-related proteins may be able to inhibit complement more efficiently than the other FH-binding proteins, thus compensating for the lack of other FH-binding proteins. The interactions of FhbA-related proteins with other complement proteins (Table 4) might also affect overall regulation of complement. It cannot be excluded that other, yet unknown, FH-binding proteins exist in relapsing fever spirochetes, or that some other complement evasion mechanisms, like binding of C4BP [57] or C1-inhibitor [58] provide enhanced protection.

Five FH-binding FhbA-related proteins have been shown to mediate serum resistance. *B*. *hermsii* strain YOR, which expresses BhFhbA, is more resistant to serum and causes more persistent infections in mice when compared with strain REN, which naturally lacks FhbA [52]. Expression of three FhbA-related proteins (BhCRASP-1[59], HcpA [33], BpcA [32]) in the serum-sensitive strain *Bo*. *burgdorferi* B313 led to increased serum survival of the mutant strain. In addition, *B*. *miyamotoi* CbiA established serum resistance when expressed in serum sensitive *Bo*. *garinii* strain G1[34]. Surprisingly, an *fhbA* knockout strain created from *B*. *hermsii* strain YOR retained resistance to complement *in vitro* and in mice, even though the strain did not express FhbA nor bound FH [55].

To analyze the effect of BhFhbA on serum mediated killing, we expressed BhFhbA and BhFhbA mutant Phe154Ala on the surface of a serum-sensitive laboratory strain of *E*. *coli*. In that environment wild-type BhFhbA, but not the mutant, protected bacteria from complement killing (Fig 3E). The binding mechanism suggested by our structure thus appears to be important also in a more physiological context. Similar results were obtained from the assay, where we incubated live *B*. *hermsii* borrelia in whole blood and measured complement activation (Fig 3E). Wild-type BhFhbA competed with *B*. *hermsii* for FH whereas the Phe154Ala mutant did not.

Our results thus demonstrate that BhFhbA is also functional on the surface of *E*. *coli* and can provide protection from complement in a natural environment. Previous results from the *fhbA* knockout strain suggest that other yet unidentified mechanism(s) to prevent formation of membrane-attack complexes may exist in *B*. *hermsii* strain YOR. This is not, perhaps, surprising because pathogens typically have several mechanisms that act alone or in tandem to help the bacteria evade innate immunity.

In summary, we present here a high-resolution structure of BhFhbA, an outer-surface complement evasion mediating protein from *Borrelia hermsii*, in complex with FH19-20. We found a dozen highly homologous proteins from Lyme disease and relapsing fever spirochetes, thus identifying a new family of immune evasion proteins, which we name the FhbA-related protein family. We propose that FhbA-related proteins are important complement evasion molecules in RF borreliae, and thus represent important targets to develop tools to prevent infections caused by borreliae.

## Materials and methods

### Ethics statement

Blood samples were drawn from healthy human volunteers by trained professionals after donor review of information fact sheet and written and signed consent as approved by the Ethical Committee (decision HUS/135/2020) of Hospital district of Helsinki and Uusimaa.

#### Bacteria and sera

*Borrelia hermsii* strain HS1 was a kind gift from prof. Bergström, University of Umeå, Sweden. Bacteria were cultured in BSK-H media (Sigma-Aldrich, Darmstadt, Germany) at +33° C in 5% CO_2_ and 100% humidity and number was determined by calculation under dark-field microscopy using 40x magnification. Prior to usage bacteria were pelleted (8,000 *g* 15 min at RT) and washed 3 times with PBS (phosphate-buffered saline; 120 mM NaCl, 30 mM phosphate, pH 7.4). Blood was drawn into hirudin (Roche Diagnostics, Mannheim, Germany) tubes from healthy human volunteers after informed written and signed consent (Ethical Committee decision HUS/135/2020, Hospital district of Helsinki and Uusimaa). The plasma was isolated by centrifugation. To obtain serum the blood was drawn into serum tubes, blood was allowed to clot, after which it was centrifugated and serum collected. Serum was kept at -80° C and heat-inactivated (+56° C, 30 min) to remove complement activity.

#### Plasmid construction and mutagenesis

Wild type BhFhbA without its leader peptide (residues 44–202) from the strain *B*. *hermsii* YOR was cloned and purified as previously described [29]. YOR was chosen, as we had previous data on binding of FH to YOR [30] and we wanted to continue with the same protein to facilitate thorough analysis of the binding mechanism. FhbA-related genes from *B*. *duttonii* CR2A (wild type and F130A mutant) were synthesized at Twist Bioscience (San Francisco, USA) codon-optimised for expression in *E*.*coli* and cloned into the same vector as BhFhbA by replacing its open reading frame using NEBuilder kit (E5520S, NEB) according to the manufacturer’s instructions. Sequences were verified by Sanger sequencing in both forward and reverse directions.

Alanine point mutations to the BhFhbA gene were generated by site-directed mutagenesis. All primers used (listed in S2 Table) contained a minimum 15 bp complementary region for the BhFhbA gene on both the 5’ and 3’ ends, with the site of mutation located roughly in the centre. The PCR reaction was carried out using the standard protocol for KAPA HiFi HotStart Ready Mix (KAPABiosystems). An aliquot of the PCR reaction was treated with FD-DpnI restriction enzyme (Thermo Fisher Scientific, MA, US) prior to transformation into XL10 Gold chemically competent cells (Agilent) that were grown overnight on Luria Bertani plates, after which single colonies were picked for plasmid extraction and sequencing.

Plasmids for expression of BhFhbA and BhFhbA/Phe153Ala in *E*. *coli* using the AIDA-system [44] were cloned into pAIDA1 plasmid, which was a gift from Gen Larsson (Addgene plasmid # 79180; http://n2t.net/addgene:79180; RRID:Addgene_79180). The coding sequences of wild type and Phe153Ala mutant were PCR-amplified with primers containing overlap regions that match the pAIDA1 insert site (S2 Table), using a standard protocol for KAPA HiFi HotStart Ready Mix (KAPABiosystems). PCR products were then purified and assembled with PCR-linearized pAIDA1 plasmid using NEBuilder kit (E5520S, New England Biolabs, MA, US) according to the manufacturer’s instructions.

#### Expression and purification of proteins

Wild-type FH19-20 and FH5-7 were cloned, expressed in *Pichia pastoris* and purified using heparin affinity column as previously described [35]. C3b, factor I and Factor H were from Comptech, TX, USA and ApoE3 from Aviva biosystems (CA, US). Sequence verified plasmids of wild type and mutated FhbA genes were transformed into *E*. *coli* BL21 (DE) (Invitrogen, UK) for protein production. The cultures were grown at +37° C, 220 rpm shaking to an OD_600_ of 0.4 in LB-media with 100 μg/ml ampicillin and protein production was initiated by adding 0.2 mM isopropyl-β-d-thiogalactopyranoside (IPTG). Culturing was continued for 3–4 hours, and bacteria collected by centrifugation at 9,000 *g* for 20 min (+4° C). Pellets were suspended in 20 mM Tris-HCl, 150 mM NaCl, pH 7.5, flash-frozen in liquid nitrogen and stored at -70° C.

Pellets were thawed in RT water bath and lysed using an Emulsiflex C3 at +4° C (3 times at 15,000 psi pressure). The lysates were centrifuged for 30 min at 42,000 x *g* at +4° C. Supernatants were incubated with Ni-NTA agarose beads (Qiagen) for one hour at +4° C shaking. After three washes (20 mM Tris-HCl, 25 mM imidazole, 150 mM NaCl, pH 8.0) proteins were eluted with 300 mM imidazole in 20 mM Tris-HCl, 150 mM NaCl, pH 8. Eluates were concentrated with Amicon Ultra centrifugal concentrators (10 kDa cutoff) and run on ÄKTA HiLoad 16/60 Superdex 200 gel filtration column equilibrated with PBS. The largest peak was collected and concentrated using Amicon Ultra centrifugal concentrators (10 kDa cutoff). Protein concentration was measured with a NanoDrop (Thermo Fisher Scientific, MA, US).

### Crystallization, data collection and structure analysis

The BhFhbA:FH19-20 complex was crystallized at 20° C at a 1:1 molar ratio by mixing defined amounts of the two purified proteins together prior to crystallization. The reference concentration was 8 mg/ml of BhFhbA and the crystallization trials used sitting drop vapour diffusion in 200 nl drops (100 nl of protein complex solution and 100 nl of well solution). Plate-like crystals first appeared in the Helsinki Random Screen 1 (HR1) as well as in the Helsinki Complex screen. Hit conditions were manually optimised by preparing hanging drops with 2 μl of protein and 2μl reservoir. Harvestable 3D crystals appeared within two weeks at 20° C from the following conditions: 0.1 M MES pH 6.7, 0.2 M Ammonium Sulfate, 20% (v/v) PEG 4000. The crystals were picked, cryoprotected with 20% (v/v) ethylene glycol in the mother liquid, and flash frozen in liquid nitrogen for storage and transportation to the synchrotrons. Diffraction data were collected at European Synchrotron Radiation Facility (ESRF, Grenoble, France) on beamline ID23-2, at 100 K on a Pilatus3 detector (Dectris). A full 360° dataset (3600 images) was collected at an oscillation angle of 0.1°, transmission energy of 18.3%, with 0.2 s exposure time per frame. Data were merged and scaled using X-ray Detector Software (XDS) and autoPROC. Molecular replacement was done using Phaser from the Phenix package with the published structure of FH19-20 (PDB 4J38) as a search model. An initial structure of BhFhbA was built manually into clear electron density. Several cycles of manual building using Coot [36] and refinement with BUSTER [37] resulted in a final structure with R_work_/R_free_ = 0.19/0.24. We used ACHESYM software for reindexing and redefining the origin. Coordinates and structure factors were deposited to the PDB with accession code 6ZH1.

#### Binding assays

Binding affinities between FH19-20 or FH (from Complement Technologies, US) and wild type and mutants of FhbA proteins was determined using Microscale Thermophoresis (MST) with a Monolith NT.115 instrument (Nanotemper Technologies, Germany). FH19-20 and FH were labelled with RED-tris-NTA dye (Nanotemper Technologies, US) in PBS according to the manufacturer’s instructions. 10 μl of 300 nM labelled protein was mixed with 10 μl of ligand in PBS/0.025% Tween-20, the mixture loaded into standard treated capillaries (Nanotemper Technologies, US) and thermophoresis was measured at 22° C for 22–30 s with 20% LED power and 20%/60% infrared laser power. Three independent measurements were made, and results were analysed using the MO. Affinity Analysis software version 2.1 (Nanotemper Technologies, US). For gel filtration 100 μl of proteins (20 nmole) were eluted individually and in combination with FH19-20 on a Superdex 200 Increase 10/300 GL column attached to an ÄKTA (GE-healthcare) with PBS buffer at +4° C. 1 ml fractions from each run were collected and subjected to SDS-PAGE analyses on TGX gradient (4–20%) precast mini-gels (Biorad, CA, US), which were fixed, stained with QC Colloidal Coomassie Stain (Biorad), and proteins were visualized using Image Lab (Biorad). For high salt experiments, PBS buffer supplemented with 500 mM NaCl was used.

#### Western blotting

500 ng of purified BhFhbA or mutant proteins were subjected to nonreducing SDS-PAGE and transferred onto nitrocellulose membranes. Nonspecific binding was blocked with 3% fat-free milk in PBS for 1 hour at 22° C. The membranes were incubated with purified FH19-20 (10 μg/ml in 3% fat-free milk in PBS) for 12 hours at +4° C. After three washes (PBS, 0.05%Tween) membranes were incubated with goat polyclonal anti-FH antibody (Quidel, CA, US) at a 1:2000 dilution in 3% fat-free milk in PBS for 3 hours at RT. After three washes with PBS/0.05% Tween a HRP-conjugated rabbit donkey anti-goat antibody (Merck, NJ, US) was added at a dilution of 1:2000, and the membranes were incubated at 22° C for 1 hour. After three washes the bound antibodies were detected by enhanced chemiluminescence. Mutant proteins were also detected using mouse anti-His-antibody (Merck, NJ, US) at a 1:2000 dilution and HRP-conjugated secondary rabbit-anti-mouse antibody (1:5000 dilution) to detect his-tagged mutant proteins.

#### Cofactor-activity assay

1 μg/ml of BdFhbA was coated on 96-well ELISA-plates (Thermo Scientific, MA, US) in 0.5 M NaHCO₃ pH 9.6 for 12 hours +4° C. Wells were washed 3 times with 300 μl PBS, after which heat-inactivated NHS (10% in PBS) or 16 nM of FH was added into the wells, which were then incubated at +37°C for 60 min and washed 3 times with PBS. 50 nM of C3b and 40 nM of Factor I were added, after which wells were incubated for 60 min at +37° C. As a control, C3b was incubated with Factor I or with factor I and Factor H (16 nM). Samples from wells were collected and run on SDS-PAGE under reducing conditions. and transferred onto nitrocellulose membranes. Nonspecific binding was blocked with 3% fat-free milk in PBS for 1 hour at 22° C, after which monoclonal anti-C3c antibody (Quidel, San Diego, CA, USA) (1:2000 in 3% fat-free milk in PBS) was added and membrane was incubated for 12 hours at +4° C. After three washes with PBS/Tween, 0.5%, bound primary antibody was detected with HRP-conjugated rabbit anti-mouse antibody (Jackson ImmunoResearch, Cambridgeshire, UK) (1:500 dilution in 3% fat-free milk in PBS) for 60 min. After three washes, the bound antibodies were detected by enhanced chemiluminescence.

#### Complement activation assays

80 μl blood with 5 x 10^5^ *B*. *hermsii* bacteria were incubated at 37° C 5% CO_2_ atmosphere for 30 min in the presence of BhFhbA wild type or BhFhbA Phe154Ala mutant (9 μM concentration). Complement activity was stopped by adding 50 mM EDTA (ethylenediaminetetraacetic acid). Plasma was separated from the blood cells by centrifugation at 600 × *g* for 10 minutes. diluted 1:30 and analyzed by SC5b 9 Enzyme Immunoassay according to the manufacturer’s instructions (MicroVue SC5b 9 Plus Enzyme Immunoassay, Quidel, CA, US).

#### Serum sensitivity assay of *E*. *coli*

Transformed *E*. *coli* strains were grown o/n at +37° C on a shaker at 200 rpm in LB media containing 5 μg/ml chloramphenicol, diluted to 0.1 OD600, and expression of proteins was induced with 1mM IPTG. For western blot analysis of the outer membrane localization of the His-tagged proteins cells were collected and the fractionations were done as published previously [45]. After 3 hours growth, bacteria were pelleted (3,000 *g* 10 min at RT) and diluted into veronal buffered saline (VBS) so that each reaction contained 10^5^ bacteria with 10% NHS with 5 mM MgCl_2_ and 10 mM EGTA. In the control, serum was inactivated with 10 mM EDTA in VBS. The samples were diluted 1:1 into ice cold PBS at time point 0 and after 15 min incubation at +37° C. To obtain suitable amounts of bacteria, 50 μl from the assays and serial dilutions (1:10, 1:100) were plated onto Luria-Bertani plates containing 5 μg/ml chloramphenicol. After growth at +37° C, colonies were counted and the percentage survival (number of bacteria after exposure to serum divided by bacteria at time point zero x 100).

#### ELISA assays

1 μg/ml of BhFhbA or BdFhbA were coated onto 96-well ELISA-plates (Thermo Scientific, MA, US) in 0.5 M NaHCO₃ pH 9.6 for 12 hours +4° C. Wells were washed 5 times with 300 μl PBS and blocked (60 min RT). Buffers used in all dilutions and washes were 0.5% BSA in PBS for monoclonal antibodies and 0.5% Tween in PBS for polyclonal antibodies. Serial dilutions of FH19-20 or FH5-7 were prepared on non-adherent plastic plates. 100 μl pf primary monoclonal antibody VIG8 [60] against domain 20 of FH (1 μg/ml) or polyclonal goat-anti FH at a 1:2000 dilution was added and plates were incubated 60 min at +37° C. After 5 washes, secondary HRP-conjugated anti-mouse or anti-goat antibody (both from Jackson ImmunoResearch, Cambridgeshire, UK) were added at a 1:5000 dilution and incubated for 60 min at +37° C. After five washes the substrate, *o*-phenyl-diamine diluted in H_2_O and supplemented with 0.04% H_2_O_2_, was added. After a 15 min incubation at +22° C the reaction was stopped by adding 50 μl of 2M H_2_SO_4_ per well. The absorbances were read with an ELISA-reader using a 492 nm filter.

#### Sequence analysis and protein family identification

The full sequence of the FhbA protein from *B*. *hermsii* YOR (UniprotID: W5SB08) was submitted to Position-Specific Iterated (PSI)-BLASTp search using the NCBI portal against the non-redundant protein sequences database. After each iteration, the sequences for the next round were manually selected in order to have a representative group of unique sequences from different borreliae families. It took 5–6 rounds to obtain a comprehensive list of family representatives. Increasing repetitions provided no new members. All available borreliae genomes (in total 158 genomes) were downloaded from NCBI and searched for FhbA-related proteins and other FH-binding proteins starting from the following proteins: *B*. *hermsii* FhbA (UniprotID: W5SB08), *Bo*. *burgdorferi* CcpA (CRASP-1) (UniprotID:Q66ZA0,), *Bo*. *burgdorferi* CspZ (CRASP-2) (UniprotID:O5066,), and *Bo*. *burgdorferi* OspE (UniproID: Q45001) with exonerate program [61], using parameters to match the translated genomic DNA sequence to that of the bait protein sequence. This allowed us to identify sequences at the DNA level even when the protein prediction failed, or when protein annotation was incorrect or missing in the databases. For CspZ (CRASP-2) protein, the reported genomes were missing the full-length sequence, but Rogers *et al*. reported [46] two isolated sequences, which were not deposited in any well-known database, but are nevertheless included in Table 3. Four genomes (*B*. *chilensis*, *B*. *turicatae*, *B*. *anserina*, *and B*. *recurrentis*) did not have the plasmids containing the *fhbA* gene, but the sequences had been isolated by different research groups and annotated accordingly. We combined the sequences from both searches and selected the unique representatives from each of the species for the multiple sequence alignment. In case of discrepancy or multiple matches, we selected the closest homologue to the target protein used in the search.

In this study we refer to FhbA-related sequencies from different species as homologs [62]. The accession codes for sequences used in the alignment are in the legend to Fig 4A. 3D protein homology model was obtained for each representative member using Phyre2[63] and SWISS model [64] servers freely available on-line, with our X-ray structure of BhFhbA as a template. Secondary structure elements were independently predicted using Jperd4 (PMID: 25883141) software and compared to those obtained from Phyre2 and SWISS model. Homology models were used to correct the sequence alignment by introducing gaps at relevant places, to obtain a structure-based or structure-assisted sequence alignment. This step was crucial to preserve the correct alignment of secondary-structure elements (α-helices).

#### Circular dichroism (CD) spectrometry of purified proteins

The CD spectra for the wild type and mutant FhbA-proteins were collected in 20% PBS buffer diluted with water, at 20°C, on a J-720 spectropolarimeter (Jasco MA, US), in 300 μl quartz cuvette of 0.1 cm light path length with the following parameters: continuous scanning mode with scanning speed of 20 nm/min, band width 0.5 nm, wave range 190–260 nm, data pitch 0.5 nm. All the proteins were thawed and diluted to 12.5 or 15 μM. The accumulation of 5 scans for each protein has been plotted as a single curve.

#### Statistical analysis and data fitting

*K*_d_-values were calculated from each individual experiment separately (and shown as an average in Table 2) in the MST-software with following equation

$$K_{d}\;formula\;(law\;of\;mass\;action)$$

$$f_{\left(c\right)}=\frac{unbound+(bound-unbound)}{2\times ([Fluorophore]+c+K_{d}-\sqrt{{\left([Fluorophore]+c+K_{d}\right)}^{2}-4\times [Fluorophore]\times c}}$$

For comparison of data from different experiments, data were normalised in GraphPad Prism 8 (Figs 2C and 2D, 4D and S2). Data fits shown in the figures include all data from each experiment. We used following equation available in the Prism 8 to fit the normalised data for visualisation purposes:

$$Y=\frac{Minimum\;value+(Minimum-maximum\;value)}{\left(1+10^{\left({Log}_{EC50}-X\right)}\right)}$$

To evaluate the goodness of the fit, we adjusted R-squared formula

$$R_{2}=\frac{1-(\frac{{SS}_{residuals}}{\left(n-K\right)})}{\left(\frac{{SS}_{total}}{n-1}\right)},\;where\;K=number\;of\;parameters$$

and values were typically at worst 0.55 for BdFhbA mutant, and best for BhFhbA wild type, 0.93. Typically values for fit were ~0.8.

The statistical significance between mutants was calculated using Student’s *t*-test, unpaired values, from three replicate *K*_d_ -values.

In the serum survival test statistics was calculated using SPSS software, IBM statistics from at least 4 replicates using one-way Anova supplemented with Dunnet’s post-hoc test for unequal variances.

## Supporting information

S1 Fig BhFhbA:FH19-20 interfaces in the crystal. (**a**) Two non-overlapping interaction interfaces of BhFhbA proteins with the FH19-20 fragment are observed in the crystal. (**b**) Biological interface. (**c**) Crystal symmetry interface formed because of packing of protein molecules in the crystal.
(TIFF)

S2 Fig Analysis and visualization of multiple binding molecules to the FH19-20 fragment. **(a)** FH19-20 (blue) is represented with many biological molecules that were reported to bind FH19-20. The binding area of FhbA, OspE and sialic acid overlap. The C3d binding region on FH19 does not overlap with the secondary interface observed in the FhbA:FH19-20 co-crystals. **(b)** Sialic acid makes multiple interactions with W1183 on FH, analogous to BhFhbA.
(TIFF)

S3 Fig Western blot analysis of wild type and mutant BhFhbA binding to FH19-20. (**a**) His-tagged BhFhbA proteins were run on SDS-PAGE, the proteins transferred to a membrane, and the transferred proteins were detected by anti-His–antibody. (**b**) BhFhbA mutant proteins were separated by SDS-PAGE and transferred to the membrane, which was incubated with purified FH19-20. The bound FH19-20 was subsequently detected by a polyclonal anti-FH-antibody.
(TIFF)

S4 Fig Binding of wild type and mutant BhFhbA to FH19-20. (**a**) Raw data from the MST measurements for wild type and mutant BhFhbA proteins. (**b**) Comparison of mutants and wild-type for FH19-20 binding showing only the mean values of all measurements
(TIFF)

S5 Fig Complex formation between FH19-20 and BhFhbA. (**a**) Gel filtration elution profiles of FH19-20 and *B*. *hermsii* FhbA proteins (wild type and F154A mutant) alone, as well as when mixed with each other prior to loading on the column. The assay was done at physiological salt (PBS) and at high salt conditions (PBS + 500 mM NaCl). (**b**) SDS-PAGE analysis of the corresponding elution fractions. Wild type BhFhbA shifts the mobility of FH19-20, but the mutant does not.
(TIFF)

S6 Fig Interaction measurements of wild type and mutant BhFhbA to full-length FH. (**a**) MST binding curves for wild type and mutant BhFhbA proteins with full-length FH (**b**) Competition MST experiments of binding between BhFhbA wild type and full-length FH with no inhibitor, FH5-7 fragment and FH19-20 as an inhibitor.
(TIFF)

S7 Fig Recombinant wild type and mutant proteins are properly folded. (**a**) Circular dichroism (CD) measurements of *B*. *hermsii* and *B*. *duttonii* wild type and mutant FhbA proteins, and (**b**) their corresponding High Tension (HT) voltage traces. The spectra show that all proteins maintain their secondary structure elements (helices).
(TIFF)

S8 Fig Identity and similarity between FhbA proteins from different species. Pairwise alignment scores for identity (in green) and similarity (in yellow) between all aligned sequences shown in the Fig 4. Similarities were calculated using the BLOSUM62 matrix (with threshold equals 1), implemented in the Geneious program.
(TIFF)

S9 Fig Complex formation between FH19-20 and *B*. *duttonii* FhbA proteins. (a) Gel filtration elution profiles of FH19-20, and *B*. *duttonii* FhbA proteins (wild type and F130A mutant) alone, and when mixed with each other prior to loading on the column. The assay was done at physiological salt (PBS). (b) SDS-PAGE analysis of the corresponding elution fractions. Wild type BdFhbA shifts the mobility of FH19-20, but the mutant does not.
(TIFF)

S10 Fig Interaction measurements of wild type and mutant BdFhbA to full-length FH. MST binding curves for wild type and mutant BdFhbA proteins with full-length FH.
(TIFF)

S11 Fig Mapping mutations from Hovis *et al*. [54] onto the structure of the BhFhbA:FH19- 20 complex. Six mutants of BhFhbA with reduced or no binding to FH16-20 fragment were selected for analysis. (a) A total of 11 substitutions were mapped on the structure: seven are in the hinge area of the active binding loop (blue), and four are in other places (green). (b) Effects of mutations on FH19-20 binding [54]. (c) Selected examples of how a mutation can disrupt favorable interactions (indicated by dashed lines), and hence affect the binding of FH19-20 to BhFhbA.
(TIFF)

S1 Table Hydrogen bonds between FH20 and OspE and FH20 and BhFhbA. Distances (Å) are from the PISA-server (Krissinel and Henrick, 2007 [39]) and data from binding inhibition assays from (Meri *et al*. 2013 [30]). In biochemical assays effect of mutant protein to binding of radiolabeled FH19-20 to BhFhbA/OspE was measured, thus in regard to those results the table has no distances. For example (italics), mutation of Trp^1183^ to alanine decreased binding of FH19-20 to FhbA and OspE (grey marking) and the PISA server found a hydrogen bond between OspE Asn^77^ (Nδ2) and FH Trp^1183^ (O) with a length of 3.08 Å.
(DOCX)

S2 Table A list of primers designed for FhbA from B.hermsii. (DOCX)

S3 Table Analysed available genomes for FhbA-related, CspA/CRASP-1, CspZ/CRASP-2 and OspE genes in *Borrelia*. (DOCX)
