# Supplementary figures and images for: Mechanism of Borrelia immune evasion by FhbA-related proteins
Source: PLoS Pathog. 2022 Mar 18;18(3):e1010338. doi: 10.1371/journal.ppat.1010338 (PMC8967061; doi:10.1371/journal.ppat.1010338)

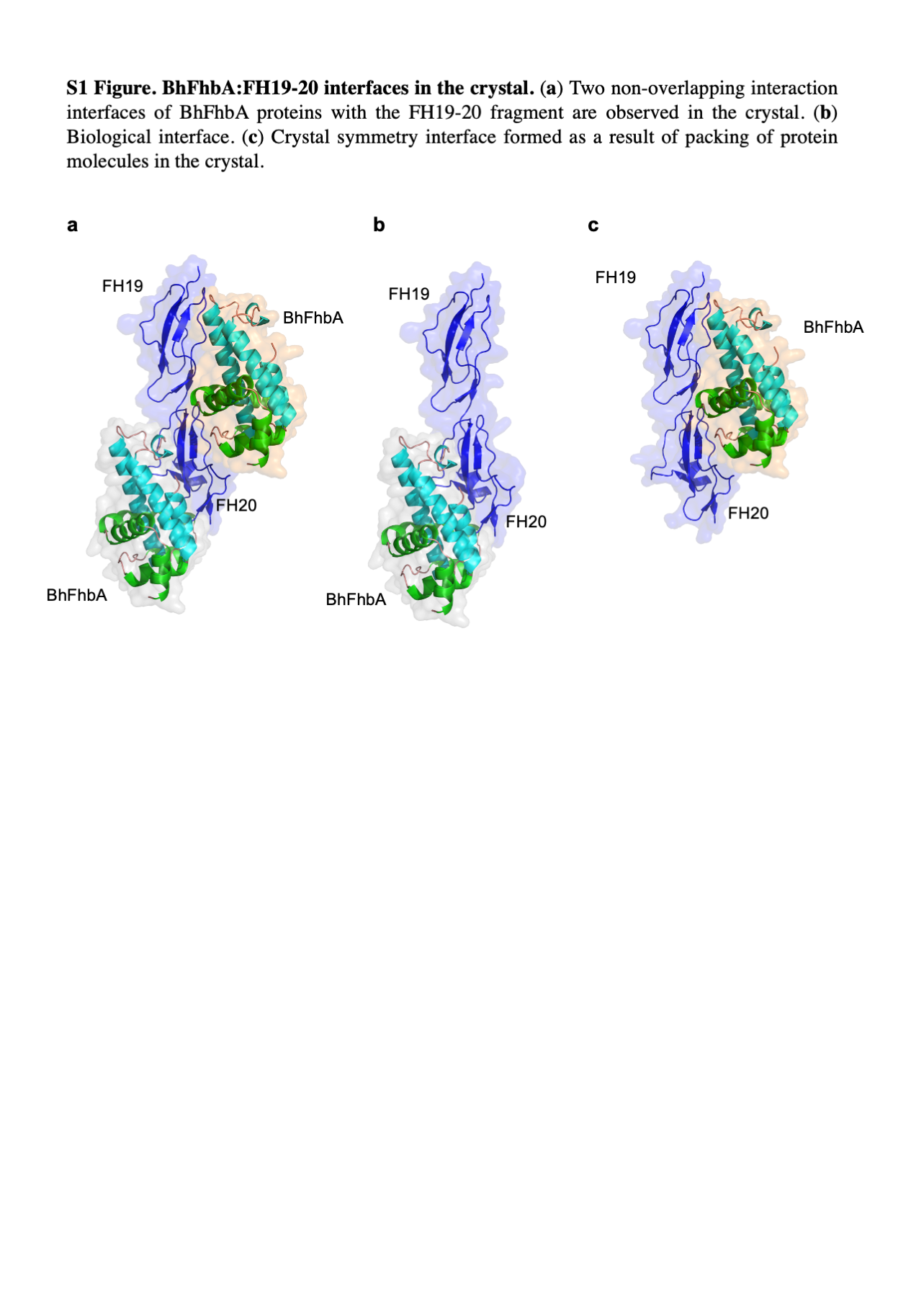

Supplement: S1 Fig — (a) Two non-overlapping interaction interfaces of BhFhbA proteins with the FH19-20 fragment are observed in the crystal. (b) Biological interface. (c) Crystal symmetry interface formed because of packing of protein molecules in the crystal. (TIFF) [file ppat.1010338.s001.tiff]

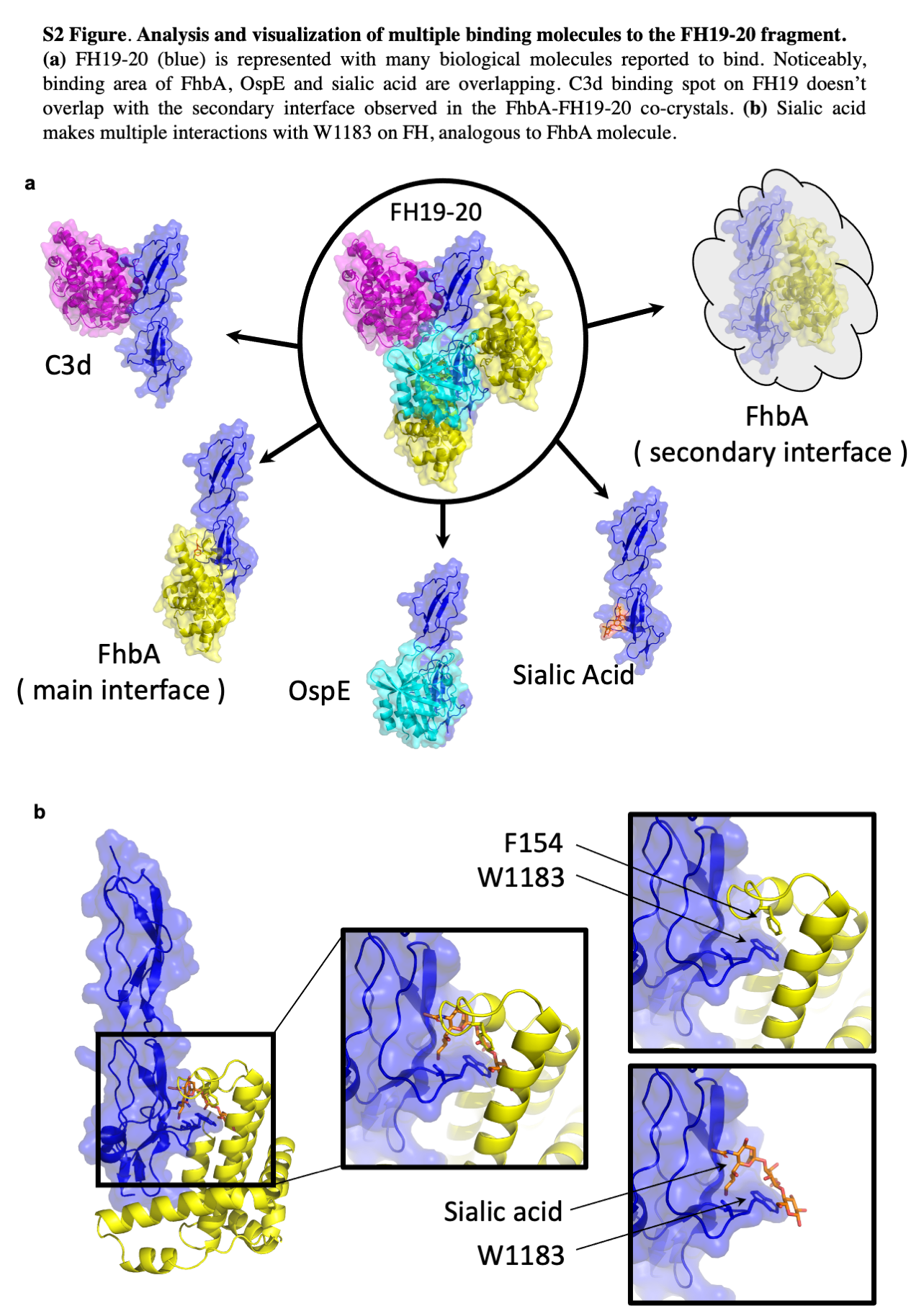

Supplement: S2 Fig — (a) FH19-20 (blue) is represented with many biological molecules that were reported to bind FH19-20. The binding area of FhbA, OspE and sialic acid overlap. The C3d binding region on FH19 does not overlap with the secondary interface observed in the FhbA:FH19-20 co-crystals. (b) Sialic acid makes multiple interactions with W1183 on FH, analogous to BhFhbA. (TIFF) [file ppat.1010338.s002.tiff]

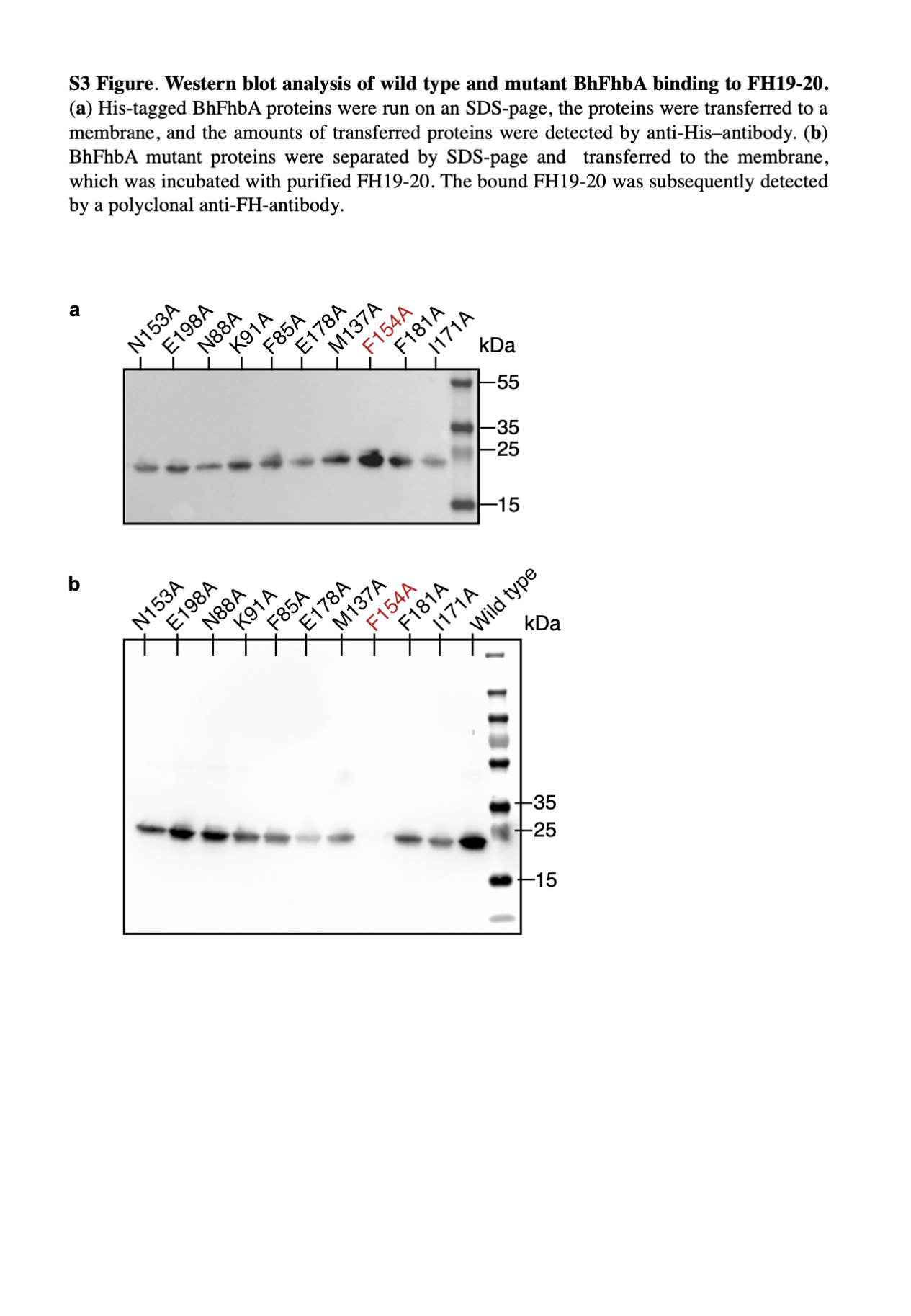

Supplement: S3 Fig — (a) His-tagged BhFhbA proteins were run on SDS-PAGE, the proteins transferred to a membrane, and the transferred proteins were detected by anti-His–antibody. (b) BhFhbA mutant proteins were separated by SDS-PAGE and transferred to the membrane, which was incubated with purified FH19-20. The bound FH19-20 was subsequently detected by a polyclonal anti-FH-antibody. (TIFF) [file ppat.1010338.s003.tiff]

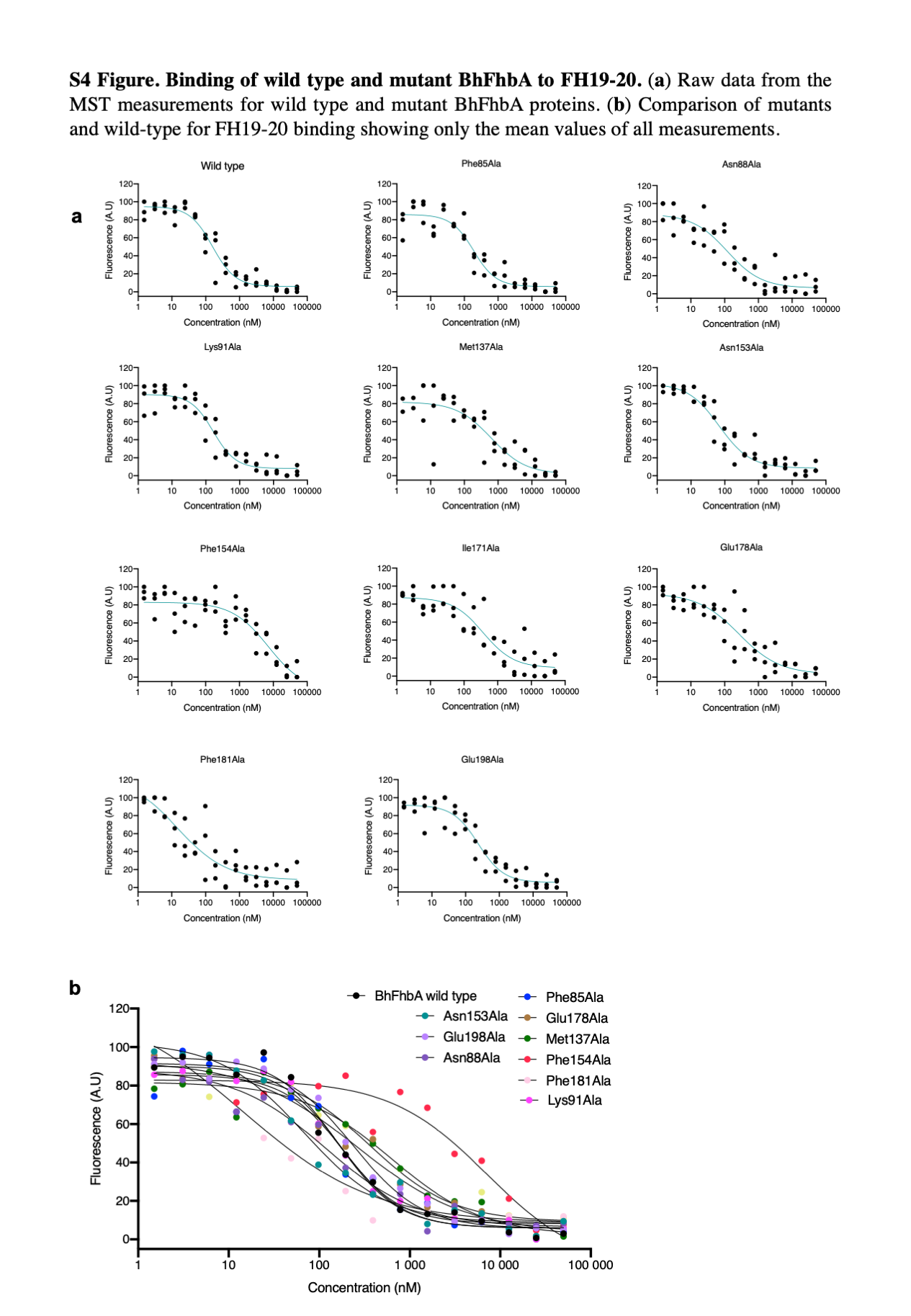

Supplement: S4 Fig — (a) Raw data from the MST measurements for wild type and mutant BhFhbA proteins. (b) Comparison of mutants and wild-type for FH19-20 binding showing only the mean values of all measurements (TIFF) [file ppat.1010338.s004.tiff]

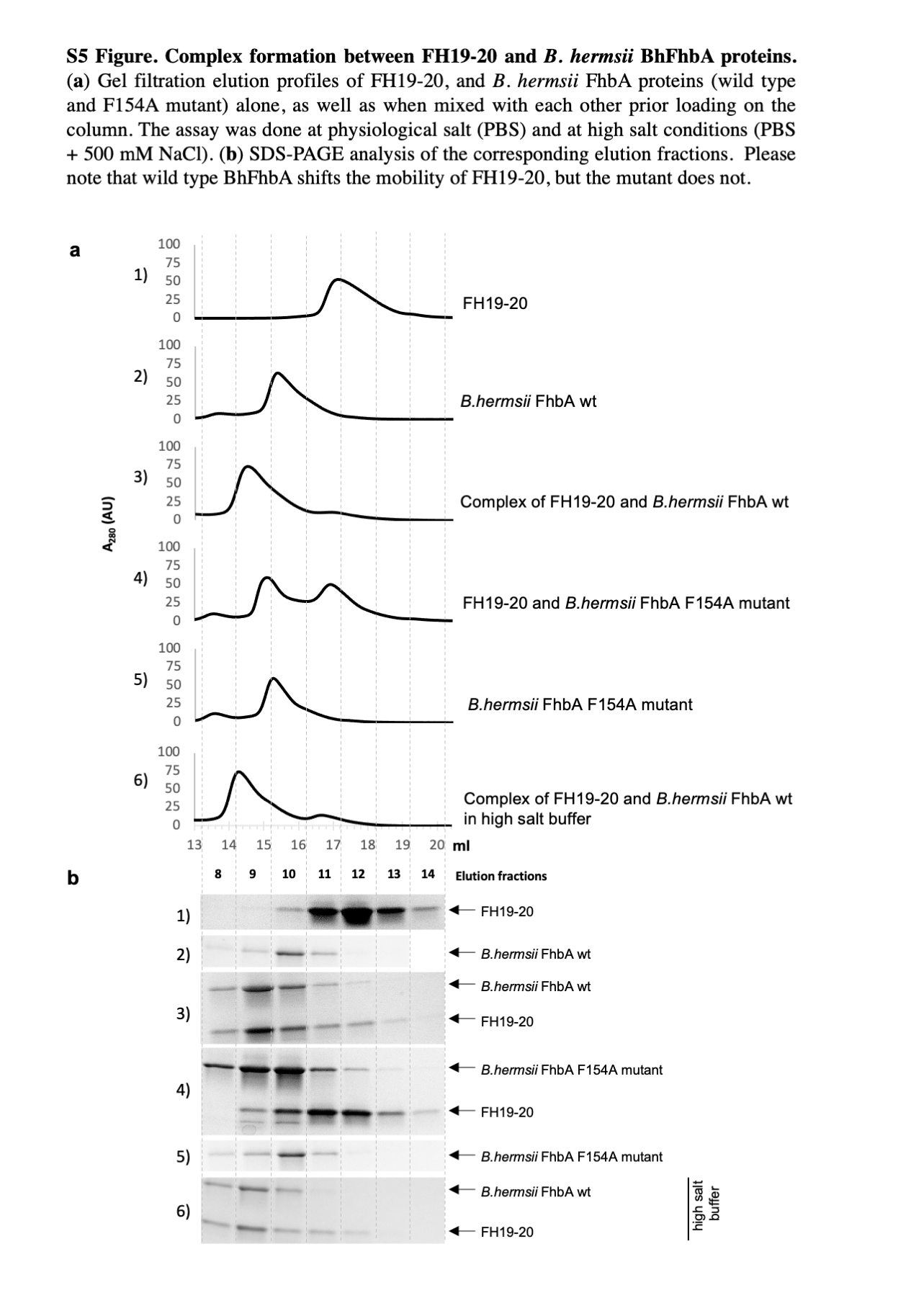

Supplement: S5 Fig — (a) Gel filtration elution profiles of FH19-20 and B. hermsii FhbA proteins (wild type and F154A mutant) alone, as well as when mixed with each other prior to loading on the column. The assay was done at physiological salt (PBS) and at high salt conditions (PBS + 500 mM NaCl). (b) SDS-PAGE analysis of the corresponding elution fractions. Wild type BhFhbA shifts the mobility of FH19-20, but the mutant does not. (TIFF) [file ppat.1010338.s005.tiff]

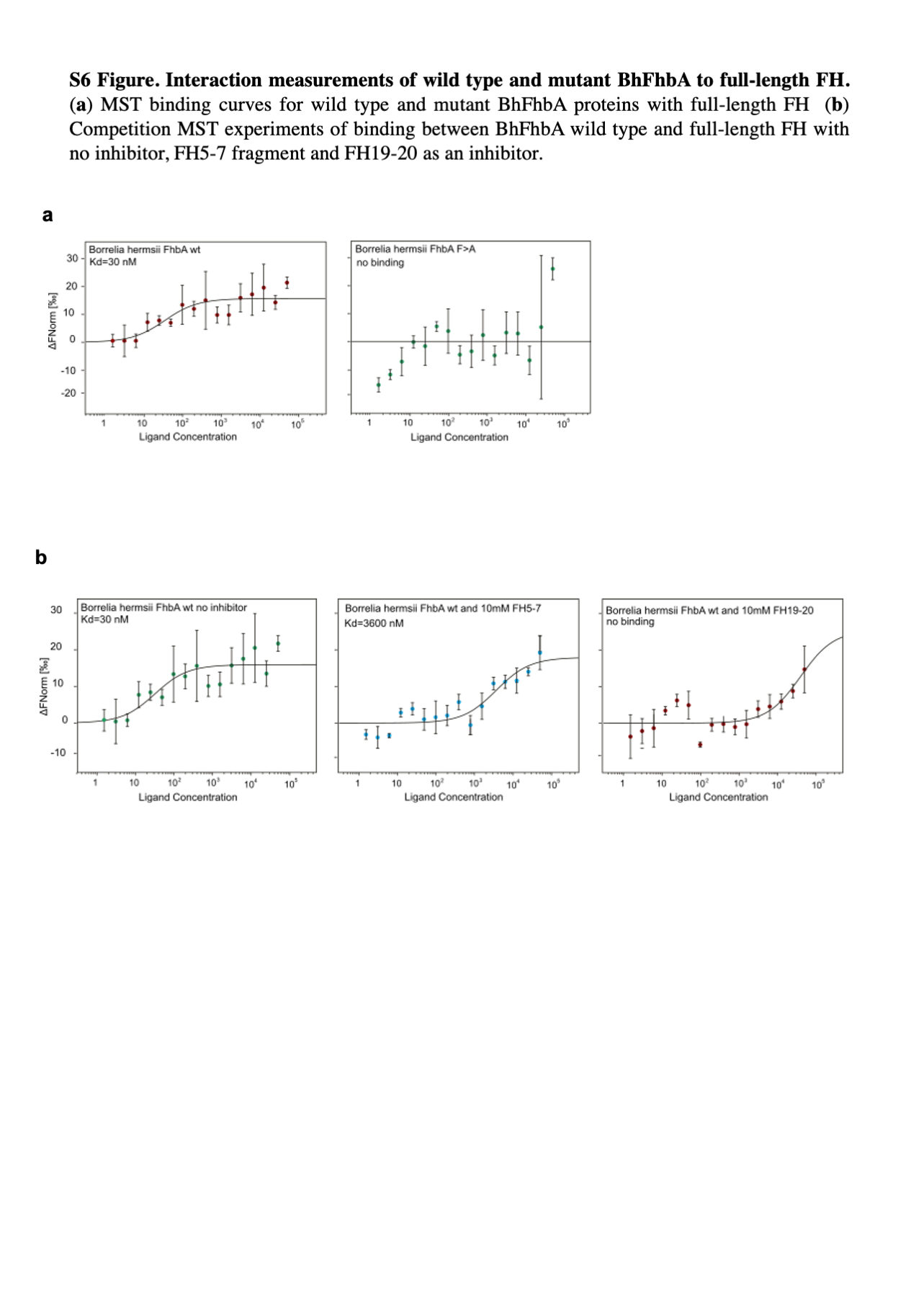

Supplement: S6 Fig — (a) MST binding curves for wild type and mutant BhFhbA proteins with full-length FH (b) Competition MST experiments of binding between BhFhbA wild type and full-length FH with no inhibitor, FH5-7 fragment and FH19-20 as an inhibitor. (TIFF) [file ppat.1010338.s006.tiff]

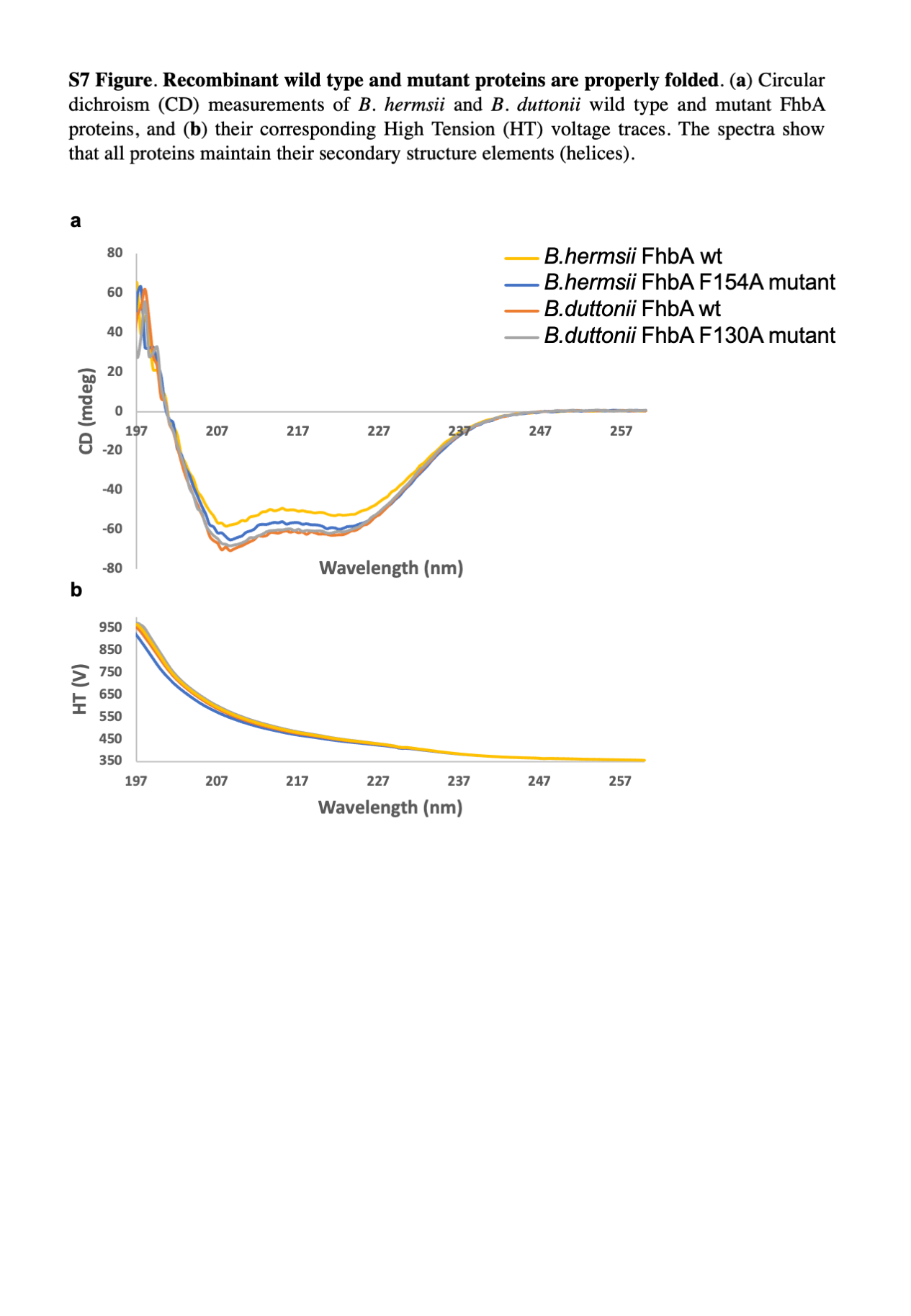

Supplement: S7 Fig — (a) Circular dichroism (CD) measurements of B. hermsii and B. duttonii wild type and mutant FhbA proteins, and (b) their corresponding High Tension (HT) voltage traces. The spectra show that all proteins maintain their secondary structure elements (helices). (TIFF) [file ppat.1010338.s007.tiff]

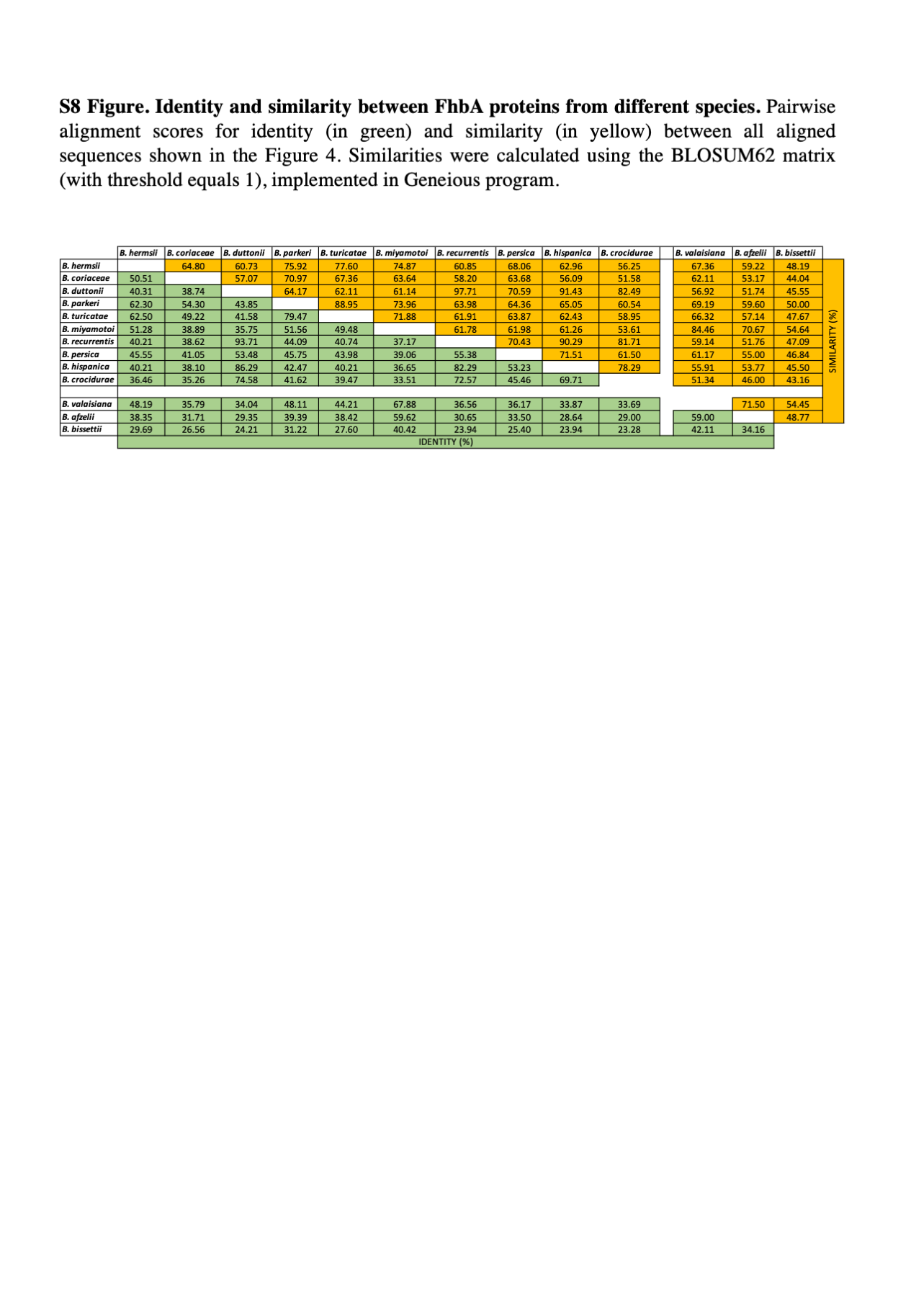

Supplement: S8 Fig — Pairwise alignment scores for identity (in green) and similarity (in yellow) between all aligned sequences shown in the Fig 4. Similarities were calculated using the BLOSUM62 matrix (with threshold equals 1), implemented in the Geneious program. (TIFF) [file ppat.1010338.s008.tiff]

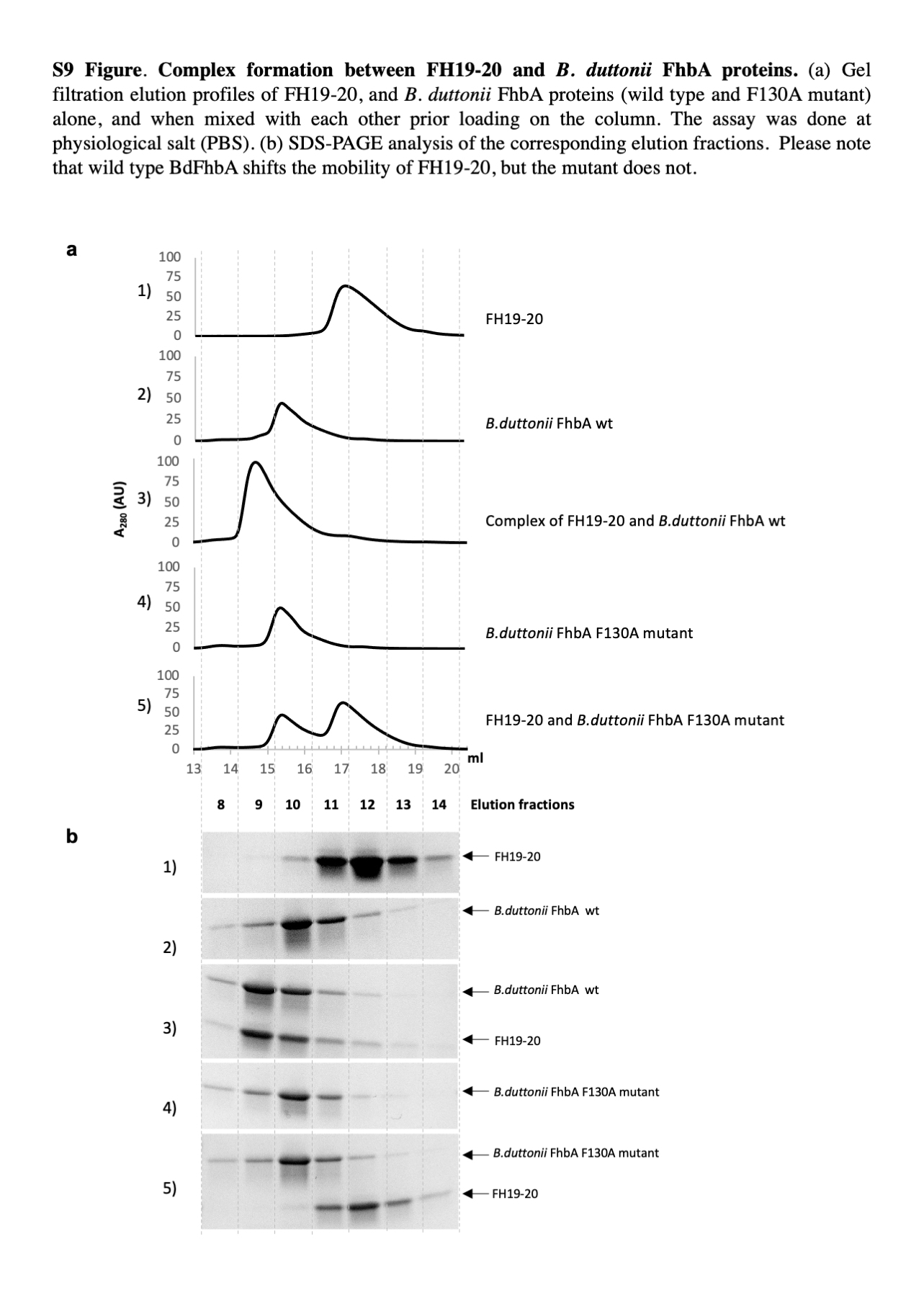

Supplement: S9 Fig — (a) Gel filtration elution profiles of FH19-20, and B. duttonii FhbA proteins (wild type and F130A mutant) alone, and when mixed with each other prior to loading on the column. The assay was done at physiological salt (PBS). (b) SDS-PAGE analysis of the corresponding elution fractions. Wild type BdFhbA shifts the mobility of FH19-20, but the mutant does not. (TIFF) [file ppat.1010338.s009.tiff]

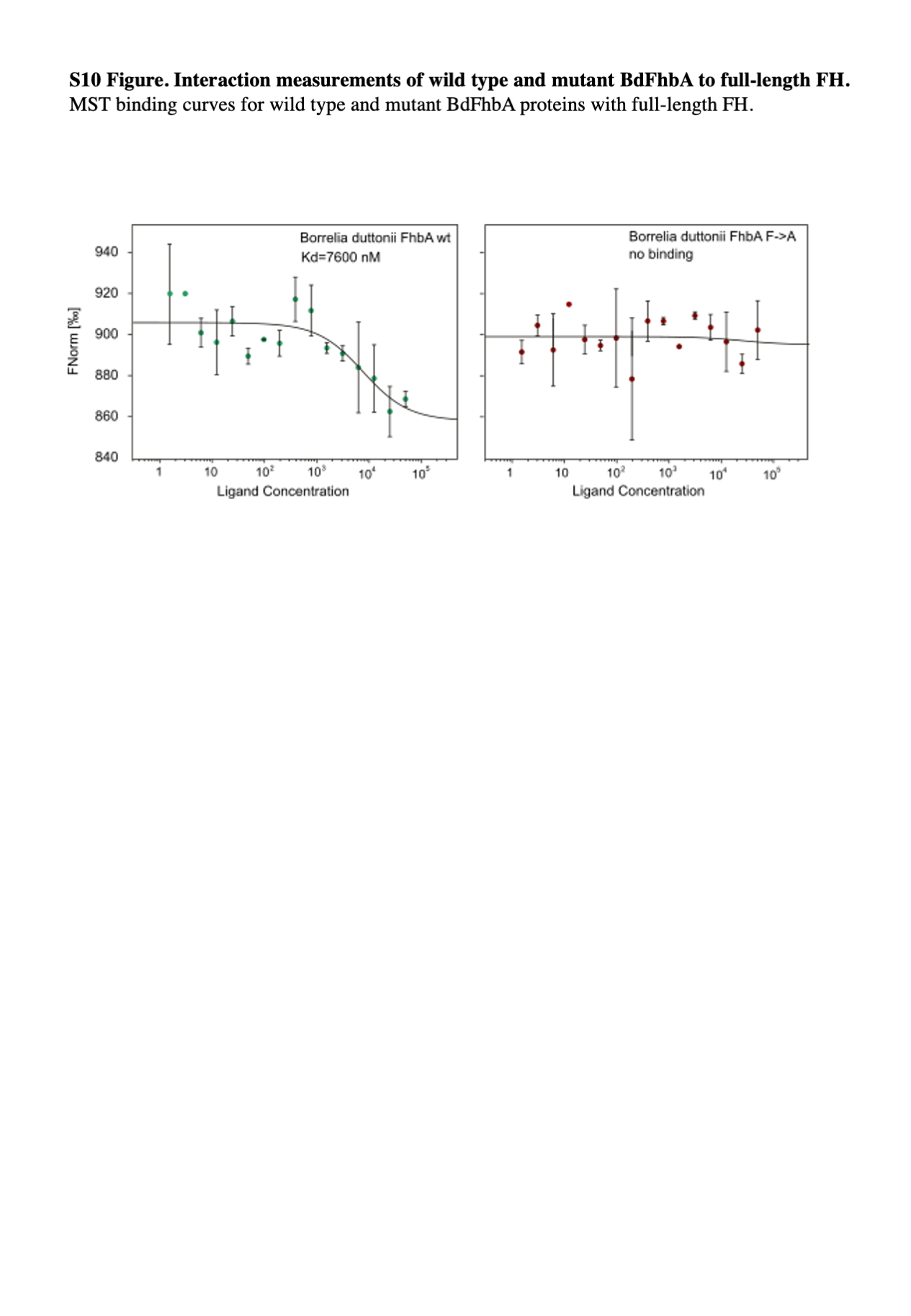

Supplement: S10 Fig — MST binding curves for wild type and mutant BdFhbA proteins with full-length FH. (TIFF) [file ppat.1010338.s010.tiff]

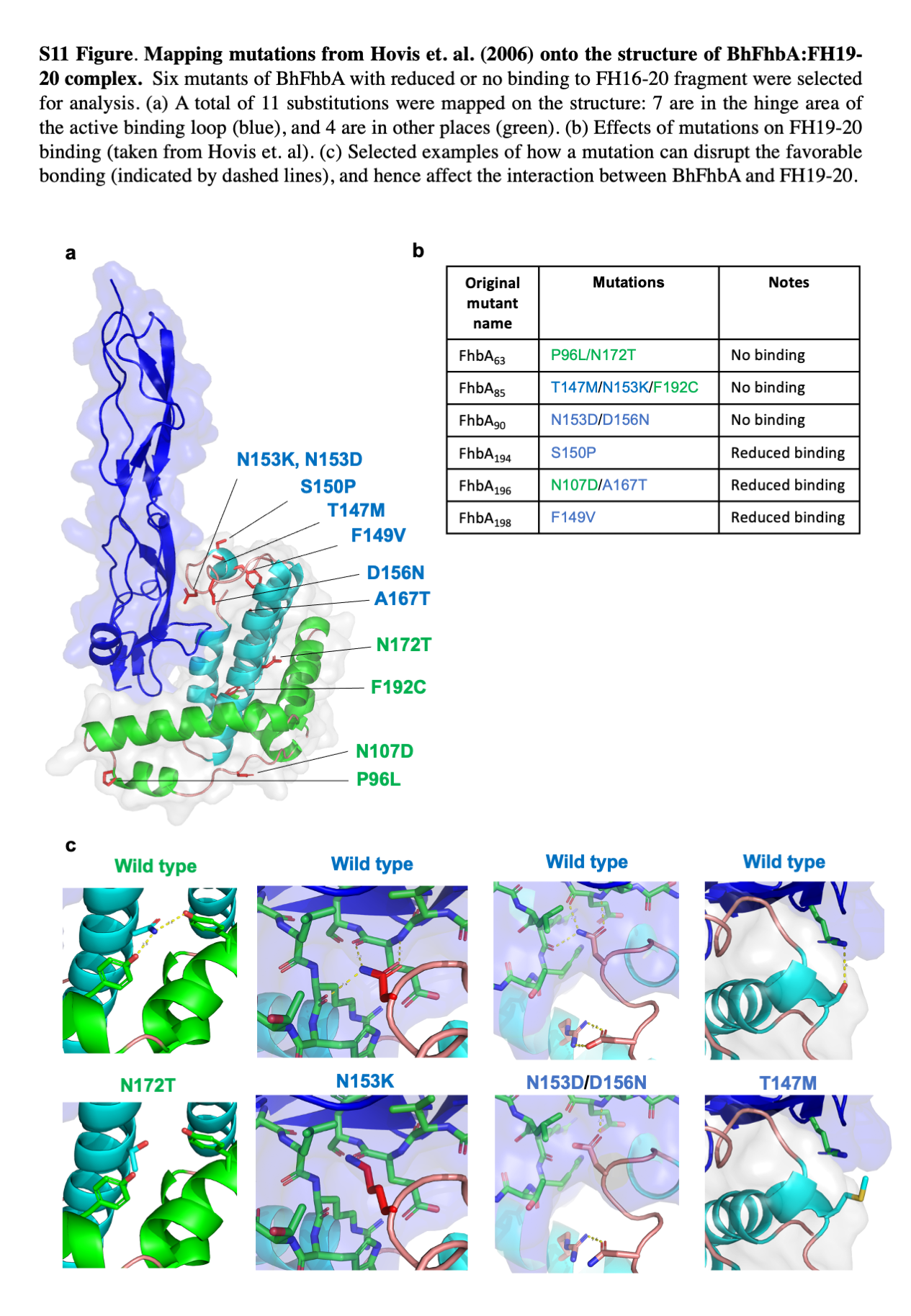

Supplement: S11 Fig — Six mutants of BhFhbA with reduced or no binding to FH16-20 fragment were selected for analysis. (a) A total of 11 substitutions were mapped on the structure: seven are in the hinge area of the active binding loop (blue), and four are in other places (green). (b) Effects of mutations on FH19-20 binding [54]. (c) Selected examples of how a mutation can disrupt favorable interactions (indicated by dashed lines), and hence affect the binding of FH19-20 to BhFhbA. (TIFF) [file ppat.1010338.s011.tiff]
