# Supplementary material for: Mechanism of Borrelia immune evasion by FhbA-related proteins
Source: PLoS Pathog. 2022 Mar 18;18(3):e1010338. doi: 10.1371/journal.ppat.1010338 (PMC8967061; doi:10.1371/journal.ppat.1010338)
Supplement: S1 Table — Distances (Å) are from the PISA-server (Krissinel and Henrick, 2007 [39]) and data from binding inhibition assays from (Meri et al. 2013 [30]). In biochemical assays effect of mutant protein to binding of radiolabeled FH19-20 to BhFhbA/OspE was measured, thus in regard to those results the table has no distances. For example (italics), mutation of Trp1183 to alanine decreased binding of FH19-20 to FhbA and OspE (grey marking) and the PISA server found a hydrogen bond between OspE Asn77 (Nδ2) and FH Trp1183 (O) with a length of 3.08 Å. (DOCX) [file ppat.1010338.s012.docx]

**S1 Table. Hydrogen bonds between FH20 and OspE and FH20 and BhFhbA.**

| FH residue | BhFhbA residue  or  BhFhbA protein | Distance (Å)  (current structure,  PDB 6ZH1) | Distance (Å)  (PDB 4J38) | OspE residue  or  OspE protein |
| --- | --- | --- | --- | --- |
| Arg^1182^ (NH2) | Glu^198^ (O) | 3.80 | 3.02 | Asp^73^ (OD2) |
| Arg^1182^ (NH2) | Tyr^199^ (O) | 3.19 | 2.97 | Glu^68^ (OE2) |
| Arg^1182^ (NH2) | Lys^201^ (O) | 2.80 | 3.75 | Thr^84^ (OG1) |
| Arg^1182^ (O) | Asn^153^ (ND2) | 2.91 | 2.67 | Arg^66^ (NH1) |
| *Trp^1183^ (O)* | *FhbA* | *-* | *3.08* | *Asn^77^ (ND2)* |
| Thr^1184^ (OD1) | FhbA |  | 3.73 | Asn^77^ (OD1) |
| Tyr^1190^ (O) | Asn^155^ (ND2) | 3.43 | - | OspE |
| Ser^1191^ (OD ) | Ser^152^(OD) | 2.70 | 2.61 | Gly^80^ (O) |
| Glu^1195^ (OE2) | Ser^152^(OH) | 2.93 | 3.70 | Gly^80^ (N) |
| Glu^1195^ (OE1) | Asn^155^ (ND2) | 2.81 | - | OspE |
| Ser^1196^ (O) | Ser^152^(N) | 2.90 | 3.02 | His^81^ (ND1) |
| Glu^1198^ (N) | Asn^153^ (OD1) | 2.87 | 2.82 | Ser^82^ (O) |
| Glu^1198^ (OE1) | Ser^148^(OG) | 2.68 | 2.90 | Thr^84^ (OG1) |
| Glu^1198^ (OE1) | Asn^77^ (ND2) | 2.77 | 2.68 | Thr^84^ (N) |
| Cys^1201^(N) | Glu^198^ (OE2) | 3.29 | - | OspE |
| Arg^1203^ (NH1) | Gln^81^ (OE1) | 2.85 | - | OspE |
| Arg^1203^ (NH2) | Glu^178^ (OE1) | 2.77 | - | OspE |
| Arg^1203^ (NH2) | Glu^178^ (OE2) | 2.67 | - | OspE |
| Tyr^1205^ (OH) | Asn^88^ (OD1) | 3.06 |  | OspE |
| Arg^1215^ (NH1) | FhbA | - | 3.71 | Thr^84^ (O) |
| Arg^1215^ (NE) | FhbA | - | 2.83 | Val^120^ (O) |
| Arg^1215^ (NH1) | FhbA | - | 3.46 | Ile^121^ (O) |
| Lys^1230^ (NH) | Glu^94^ (OE1) | 3.62 |  | OspE |

Color codes: grey: Mutation of amino acid in FH20 to alanine showed decreased binding in binding inhibition assays. yellow: Biochemical assay not performed. blue: Mutation of amino acid in FH to alanine had no effect in binding inhibition assays
