# Supplementary material for: Mechanism of Borrelia immune evasion by FhbA-related proteins
Source: PLoS Pathog. 2022 Mar 18;18(3):e1010338. doi: 10.1371/journal.ppat.1010338 (PMC8967061; doi:10.1371/journal.ppat.1010338)
Supplement: S2 Table — (DOCX) [file ppat.1010338.s013.docx]

**S2 Table. A list of primers designed for FhbA from *B.hermsii***

| **Mutant number** | **Mutation** | | **Forward (F) and reverse (R) primers** | |
| --- | --- | --- | --- | --- |
| **#1** | **Phe85Ala** | | F: GATTTAATGGAACAACAGAAAAGT**GCT**TTAGACAATTTACAGAAAAAGAAAGAAG R: CTTCTTTCTTTTTCTGTAAATTGTCTAAAGCACTTTTCTGTTGTTCCATTAAATC | |
| **#2** | **Asn88Ala** | | F: GAACAACAGAAAAGTTTTTTAGAC**GCT**TTACAGAAAAAGAAAGAAGATCC R: GGATCTTCTTTCTTTTTCTGTAAAGCGTCTAAAAAACTTTTCTGTTGTTC | |
| **#3** | **Lys91Ala** | | F: GAAAAGTTTTTTAGACAATTTACAG**GCA**AAGAAAGAAGATCCTGATTTGC R: GCAAATCAGGATCTTCTTTCTTTGCCTGTAAATTGTCTAAAAAACTTTTC | |
| **#4** | **Met137Ala** | | F: CAATTCCTTCAACAATTGCATATA**GCG**CTACAGTCTATTAAAGACGGC R: GCCGTCTTTAATAGACTGTAGCGCTATATGCAATTGTTGAAGGAATTG | |
| **#5** | **Asn153Ala** | | F: CAAGCTTTTCATCTTCA**GCT**TTCAATGACTTGCAAAATTTAG R: CTAAATTTTGCAAGTCATTGAAAGCTGAAGATGAAAAGCTTG | |
| **#6** | **Phe154Ala** | | F: CTTACAAGCTTTTCATCTTCAAAT**GCC**AATGACTTGCAAAATTTAGAG R: CTCTAAATTTTGCAAGTCATTGGCATTTGAAGATGAAAAGCTTGTAAG | |
| **#7** | **Ile171Ala** | | F: GAACGGGCATTGCAATAT**GCC**AATGGTAAATTATATGTTGAATAC R: GTATTCAACATATAATTTACCATTGGCATATTGCAATGCCCGTTC | |
| **#8** | **Glu178Ala** | | F: CAATGGTAAATTATATGTT**GCA**TACTATTTCTATATCAATGGAATTAG R: CTAATTCCATTGATATAGAAATAGTATGCAACATATAATTTACCATTG | |
| **#9** | **Phe181Ala** | | F: GGTAAATTATATGTTGAATACTAT**GCC**TATATCAATGGAATTAGCAATGCAGAC R: GTCTGCATTGCTAATTCCATTGATATAGGCATAGTATTCAACATATAATTTACC | |
| **#10** | **Glu198Ala** | | F: CAATTTTTTTGAAACTATAATG**GCA**TATTTAAAAACTTAACTCGAGTCTGGTAAAG R: CTTTACCAGACTCGAGTTAAGTTTTTAAATATGCCATTATAGTTTCAAAAAAATTG | |
|  | | | | |
| **Primers used to clone BhFhbA into pAIDA-I plasmid** | | | | |
| **Name of the primer** | | **Forward (F) and reverse (R) primers** | | **Notes** |
| BhFhbA-AIDA1 | | F: CTAGAAGCGCTGTTTCAAGGACCTGATTTATTCAATAAAAACAAAAAATTAGATGCTGAT R: ACCCTGGAAGTACAGGTTTTCAGTTTTTAAATATTCCATTATAGTTTCAAAAAAATTGTC | | Used to amplify the insert |
| pAIDA1_v | | F: GAAAACCTGTACTTCCAGGGTGAACAGAAAC R: AGGTCCTTGAAACAGCGCTTCTAGATGGTGATGGTGATGGTGGTCG | | Used to amplify the vector |
