# Supplementary material for: Mechanism of Borrelia immune evasion by FhbA-related proteins
Source: PLoS Pathog. 2022 Mar 18;18(3):e1010338. doi: 10.1371/journal.ppat.1010338 (PMC8967061; doi:10.1371/journal.ppat.1010338)
Supplement: S3 Table — (DOCX) [file ppat.1010338.s014.docx]

**S3 Table.** **Analysed available genomes for FhbA-related, CspA/CRASP-1, CspZ/CRASP-2 and OspE genes in *Borrelia*.**

| Clade | Species | The number of analysed genomes |
| --- | --- | --- |
| Lyme  disease  (LD) clade | *Borreliella burgdorferi* | 96 |
|  | *Borreliella finlandensis* | 1 |
|  | *Borreliella bissettiae* | 2 |
|  | *Borreliella mayonii* | 2 |
|  | *Borreliella spielmanii* | 1 |
|  | *Borreliella afzelii* | 5 |
|  | *Borreliella japonica* | 1 |
|  | *Borreliella garinii* | 21 |
|  | *Borreliella bavariensis* | 1 |
|  | *Borreliella valaisiana* | 2 |
|  | *Borreliella chilensis* | 1 |
| Relapsing  fever  (RF) clade | *Borrelia parkeri* | 1 |
|  | *Borrelia turicatae* | 1 |
|  | *Borrelia coriaceae* | 2 |
|  | *Borrelia hermsii* | 5 |
|  | *Borrelia anserina* | 1 |
|  | *Borrelia duttonii* | 2 |
|  | *Borrelia recurrentis* | 1 |
|  | *Borrelia crocidurae* | 2 |
|  | *Borrelia hispanica* | 1 |
|  | *Borrelia persica* | 1 |
|  | *Borrelia miyamotoi* | 8 |
